# Derivation of Ultra-High Gain Hybrid Converter Families for HASEL Actuators Used in Soft Mobile Robots

**DOI:** 10.3390/biomimetics8060483

**Published:** 2023-10-12

**Authors:** Tirthasarathi Lodh, Hanh-Phuc Le

**Affiliations:** Electrical and Computer Engineering Department, University of California San Diego, La Jolla, CA 92093, USA

**Keywords:** hybrid converter, extremely large conversion ratio, leakage inductance, high voltage breakdown, soft-charging, switched-capacitor, voltage multiplier, Dickson, ladder

## Abstract

This work proposes, analyzes, designs, and validates superior topologies of UHGH converters that are capable of supporting extremely large conversion ratios up to ∼2000× and output voltage up to ∼4–12 kV for future mobile soft robots from an input voltage as low as the range of a 1-cell battery pack. Thus, the converter makes soft robots standalone systems that can be untethered and mobile. The extremely large voltage gain is enabled by a unique hybrid combination of a high-gain switched magnetic element (HGSME) and a capacitor-based voltage multiplier rectifier (CVMR) that, together, achieve small overall size, efficient operation, and output voltage regulation and shaping with simple duty-cycle modulation. With superior performance, power density, and compact size, the UHGH converters prove to be a promising candidate for future untethered soft robots.

## 1. Introduction

The realm of robotics and actuation has witnessed a profound transformation with the advent of a remarkable innovation known as HASEL (Hydraulically Amplified Self-healing Electrostatic) actuators [1,2,3,4,5,6]. In the ever-evolving landscape of materials science and engineering, these actuators represent a convergence of groundbreaking principles, offering a tantalizing glimpse into the future of adaptable, versatile, and resilient actuation mechanisms. HASEL actuators are a testament to the ingenuity of modern engineering, blending principles from the realms of soft robotics, materials science, and electrostatics. These remarkable devices derive their inspiration from the natural world, seeking to mimic the extraordinary capabilities of biological muscles while harnessing the precision and control of traditional actuators. This pursuit has led to the development of a novel class of actuators that combine the robustness of traditional hydraulic systems with the flexibility and responsiveness of electrostatics. HASEL actuators are a promising technology with potential applications in robotics, prosthetics, haptic interfaces, and other fields due to their flexibility, self-healing capabilities, and energy efficiency [7,8,9,10,11,12,13].

HASEL actuators need an extremely high electric field (corresponding to a voltage of 4–12 kV) for their actuation. To make a lightweight driver for HASEL and support its untethered operation, a low-voltage battery source and an Ultra-High Gain Hybrid (UHGH) power conversion system are desired. A few available products [14,15,16,17] can support the required voltage range for HASEL. Unfortunately, they suffer from several critical drawbacks: (1) they are either too large or too low in power to be integrated into soft robotic systems [14,15,16,17]; (2) they are mainly constructed with bulky large-ratio coupled inductors making them incapable of scaling to large output currents while keeping a compact size for the whole system, i.e., the output current is limited to 0.5 mA [15]; (3) they all have fixed conversion ratios and do not support adjustable output voltage, and (4) their efficiency is limited to 70% [15], causing the hosting robotics systems to require power tethering or larger and heavier battery and thus reduce the system mobility. The drawbacks limit the use of these available products strictly to small-scale demonstrations with low operation speed in labs and not large-scale mobile systems in practical use. The UHGH converters in this work overcome the aforementioned challenges. The UHGH converters have higher output current capability, power density, and potentially lower cost than state-of-the-art. More importantly, the UHGH converters are capable of having adjustable output voltage with simple pulse width modulation. All these features make the UHGH converters superior candidates for the actuation of HASEL.

The salient features of the UHGH converters are (1) extremely high output voltage, which is the requirement for driving HASEL actuators; (2) extremely high conversion ratio; (3) high power and high power density, which enables the use of a low voltage battery at the input and keeps the overall driver lightweight. Many high-gain converters in the literature are compared with a UHGH converter of this work. None of the high-gain converters reported in [18,19,20,21,22,23] could drive HASEL actuators for soft robots.

To achieve the goal of driving a soft robotic system optimally and address the drawbacks of the available systems, this work makes the following important contributions:

1. This work demonstrates ∼2000× voltage gain with an output voltage of ∼7 kV and 25 W output from 3.7 V input voltage for untethered soft robots. High-gain converters reported in [18,19,20,21,22,23] has not achieved these stringent specifications.
2. Four families of UHGH converter topologies have been along with the methodical approaches to derive them. These innovative techniques can be applied to derive many more converters belonging to each family.
3. The derivation methodologies of the superior UHGH converters have been validated by presenting comparison tables containing a very compressed summary of the detailed analysis, design, and simulation results for 24 (8 HGSMEs, each blended with 3 superior CVMR structures) such useful UHGH converter configurations.
4. The salient features, pros, and cons of each UHGH converter configuration have been highlighted. The prominent advantages of the UHGH converter configurations have been highlighted. A comparative discussion summary of the UHGH converter configurations has been presented. Further validation by performing successful hardware implementation of a sample UHGH converters has been provided.
5. A detailed mathematical analysis of the CVMRs with 12 levels has been presented, which predicts the converter voltage gain well, considering the effect of loading.
6. The mathematical analysis predicts the peak, median, and lowest of all flying capacitor voltages as well as the output, even if the input voltage to the CVMR is an asymmetric square wave.
7. The work presents a detailed literature review and insightful summary of the high-gain converters and categorizes them according to their structures and features, which eventually led to the derivation of the superior UHGH converters.

Not only HASEL but the UHGH converters hold significant interest and instructional value for other electrostatic actuators. Notably, they extend their utility to some of the dielectric elastomer actuators (DEAs) [24] and electrohydrodynamic actuators (EHDAs) [25,26] as well. Examples include voltage-driven oscillating liquid systems such as the fluidic rolling robot [27] and high-performance bidirectional electrohydrodynamic pumps [28]. The rest of the paper is organized as follows. Section 2 shows a method to derive a family of UHGH converters. Section 3 presents the operation of the UHGHs. Section 4 presents the validation of the UHGHs. Section 5 presents comparative discussions on the UHGH converters. Section 6 concludes the paper. The following abbreviations are used in this manuscript: CVMR (Cap. based Voltage Multiplier Rectifier) naming convention: (T)(Y)(N)(X)(S)-CVMR, where *T* denotes the No. of switches, which can be can be either E (for Even), O (for Odd), or any integer number; Y that indicates the type of circuit can be either P (contains positive circuit), N (contains negative circuit), or D (contains Both circuits); and *N* indicates the No. of flying Cap. pairs in a complete group of TYNXD-CVMR. *N* can be any integer number; X denotes the type of filter, which can either be F (flying Cap. filter) or O (output filter); S denotes the type of device, which can either be D (Diode) or M (MOSFET). E.g., 8 Switch, positive output, Dickson, Output filter, Diode-based CVMR (8PDOD-CVMR). Special cases are N=1 (or L for (L)adder), N=2 (or M for (M)ixed), or N→∞ (D for (D)ickson). $L_{ka}$ and $L_{ma}$ are the leakage and magnetizing inductances of the winding $W_{al}$. $V_{1o}$ ($V_{1e}$) is the peak of $v_{a}-v_{b}$ ($v_{b}-v_{a}$) when the odd (even) diodes conduct for the *T*P*N*XD-CVMR.

## 2. Ultra-High Gain Converters

**Figure 1:**
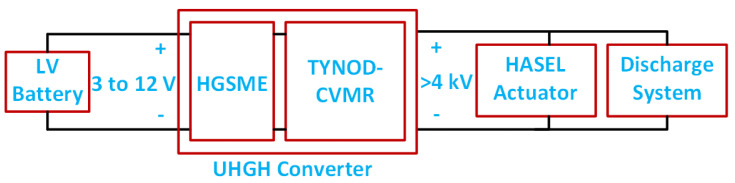
Actuation of HASEL with UHGH converters [3].

The key to the derivation of the UHGH converter with a small size and weight is to have a CVMR stage at the high-voltage output side, which can provide a voltage gain in the range of 10× (shown in Figure 1) so that the voltage gain requirement of the coupled inductor or the transformer stage is reduced. In the next subsection, the simplified idealized operation of the three diode-based optimum CVMR configurations will be presented. The following subsection will describe several combinations of a CVMR with an input-coupled inductor or transformer stage to form a UHGH converter.

**Figure 2:**
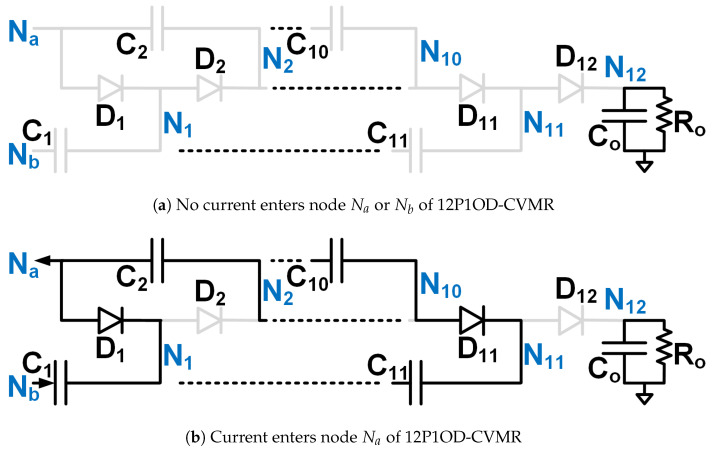

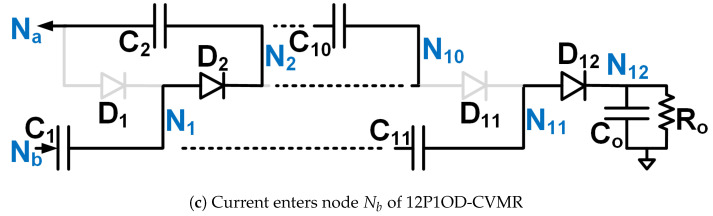
Equivalent circuits describing the operation of the 12P1OD-CVMR part.

**Figure 3:**
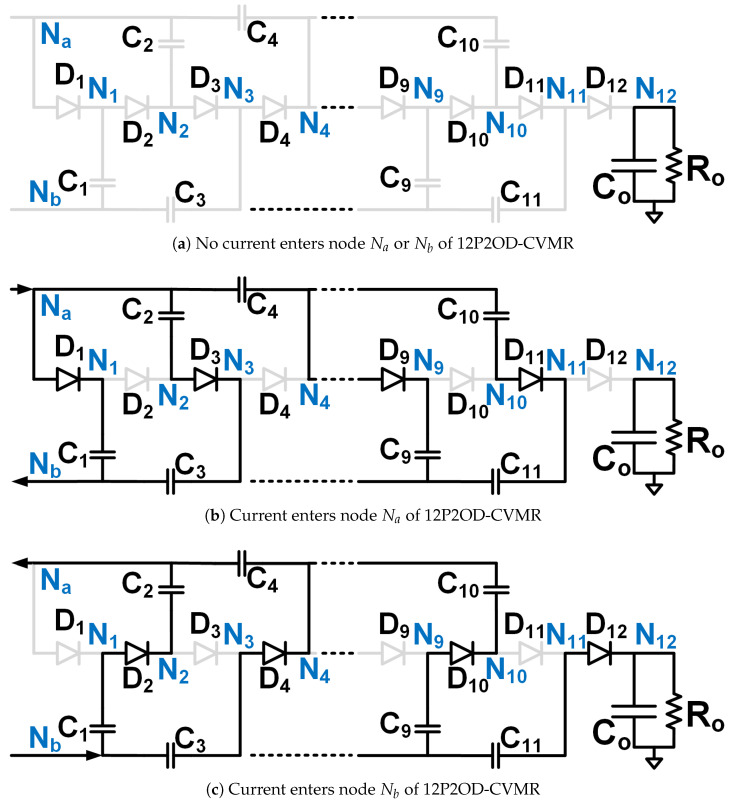
Equivalent circuits describing the operation of the 12P2OD-CVMR part.

**Figure 4:**
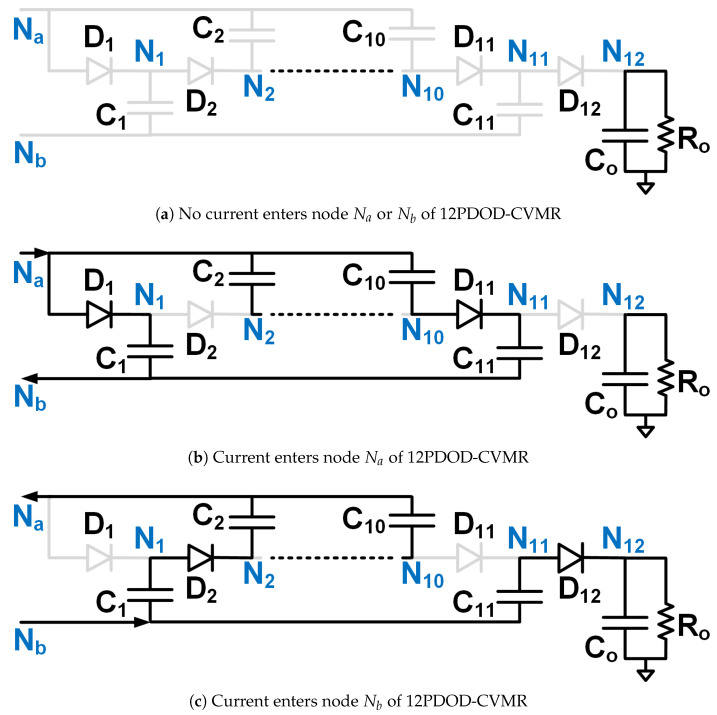
Equivalent circuits describing the operation of the 12PDOD-CVMR part.

The objective is to stay away from the immense complexity of the gate driver circuit design and make the solution simple, compact, reliable, and low-cost, which has the potential for product development in the long run. Hence, Ladder-CVMR (12P1OD-CVMR) shown in Figure 2, Mixed-CVMR (12P2OD-CVMR) shown in Figure 3, Dickson-CVMR (12PDOD-CVMR) shown in Figure 4, which can be implemented with diodes only, have been chosen. Moreover, the diodes of these topologies have the same voltage and average current ratings. They are significantly lower than the output voltage. Hence, scaling up these circuits is easy. Plus, 12P1OD-CVMR circuits are widely used in the industry for many applications.

### 2.1. CVMRs

#### 2.1.1. Operation of CVMRs

The CVMR stage employs either a 12P1OD-CVMR (Figure 2), 12P2OD-CVMR (Figure 3), 12PDOD-CVMR (Figure 4) structure with 11 flying capacitors and 12 diodes that provide an ideal no-load voltage gain of 12*X* from $V_{ab}$ to the output $V_{o}$.

When the current enters node $N_{a}$ of the CVMR (Figure 2b (12P1OD-CVMR-a), Figure 3b (12P2OD-CVMR-a), Figure 4b (12PDOD-CVMR-a)), it finds its return path through node $N_{b}$. During this period, all the odd-indexed (even-indexed) diodes $D_{1},\;D_{3},\;D_{5},\;D_{7},\;D_{9},\;D_{11}$ ($D_{2},\;D_{4},\;D_{6},\;D_{8},\;D_{10},\;D_{12}$) become forward (reverse) biased. The odd-indexed (even-indexed) capacitors $C_{1},\;C_{3},\;C_{5},\;C_{7},\;C_{9},\;C_{11}$ ($C_{2},\;C_{4},\;C_{6},\;C_{8},\;C_{10},\;C_{o}$) are softly charged (discharged). This state of CVMR (CVMR-a) is termed 12PDOD-CVMR-a for 12PDOD-CVMR (as illustrated in Figure 4b), 12P1OD-CVMR-a for 12P1OD-CVMR (Figure 2b), and 12P2OD-CVMR-a for 12P2OD-CVMR (Figure 3b).

Similarly, when the current enters node $N_{b}$ of the CVMR (Figure 2c (12P1OD-CVMR-b), Figure 3c (12P2OD-CVMR-b), Figure 4c (12PDOD-CVMR-b)), it finds its return path through node $N_{a}$. During this period, all the odd-indexed (even-indexed) diodes $D_{1},\;D_{3},\;D_{5},\;D_{7},\;D_{9},\;D_{11}$ ($D_{2},\;D_{4},\;D_{6},\;D_{8},\;D_{10},\;D_{12}$) become reverse (forward) biased. The odd-indexed (even-indexed) capacitors $C_{1},\;C_{3},\;C_{5},\;C_{7},\;C_{9},\;C_{11}$ ($C_{2},\;C_{4},\;C_{6},\;C_{8},\;C_{10},\;C_{o}$) are softly discharged (charged). This state of CVMR (CVMR-b) is termed 12PDOD-CVMR-b (Figure 4c), 12P1OD-CVMR-b for 12P1OD-CVMR (Figure 2c), and 12P2OD-CVMR-b for 12P2OD-CVMR (Figure 3c).

When there is no current entering either node $N_{a}$ or node $N_{b}$ (Figure 2a (12P1OD-CVMR-o), Figure 3a (12P2OD-CVMR-o), Figure 4a (12PDOD-CVMR-o)) all flying capacitors $C_{1}$ to $C_{11}$ stay idle approximately at their respective voltage level, except for an insignificant voltage drop due to leakage, while capacitor $C_{o}$ discharges through the output load. This state of CVMR (CVMR-o) is termed as 12PDOD-CVMR-o, 12P1OD-CVMR-o, and 12P2OD-CVMR-o for 12PDOD-CVMR (Figure 4a), 12P1OD-CVMR (Figure 2a), and 12P2OD-CVMR (Figure 3a), respectively.

For estimating the effective voltage gain of the CVMRs under loading conditions, a detailed and thorough steady-state analysis of the CVMRs is important. The estimation of the effective voltage gain of the CVMRs helps estimate the efficiencies of the converter and the actual operating conditions of devices, thus choosing proper capacitors and diodes. The peak of voltage between the CVMR input nodes $N_{a}$ and $N_{b}$ during the odd diode conduction period is denoted by $V_{1o}$. The peak of voltage between the nodes $N_{a}$ and $N_{b}$ during the even diode conduction period is denoted by $V_{1e}$. Information on these two voltages and information on the charge transferred to the output load per switching period is required to perform this analysis.

#### 2.1.2. Steady-State Analysis of CVMRs

To find the steady-state operating conditions of all flying capacitors, Kirchhoff’s Current Law (KCL) and Kirchhoff’s Voltage Law (KVL) are employed for each CVMR, both during the periods when current enters node $N_{a}$ and current enters node $N_{b}$. This results in a set of 11 equations that establishes the relationship of the charge handled by the capacitors $C_{1-11}$ and $C_{o}$ for each converter, shown in Table 1. These 11 simultaneous equations are solved to find the charge handled by the 11 flying capacitors ($Q_{C}$) in terms of the output charge per switching period (*Q*). Additionally, a set of 12 equations that relate the peak voltages ($V_{C_{z}}$) of capacitors $C_{1-11}$ and $C_{o}$ for each converter are shown in Table 2 and Table 3. Charge multiplication factor $q_{C_{z}}=\frac{Q_{C_{z}}}{Q}$ for the capacitors are listed in Table 4. Assuming a convenient implementation with all capacitors having the same capacitance *C*, substituting charge values in Table 4 into equations in Table 2, and solving them give the peak magnitude of individual capacitor voltages. The capacitor voltage median ($V_{C_{z,M}}=V_{C_{z}}-\frac{Q_{C_{z}}}{2C_{z}}$) and lowest ($V_{C_{z,L}}=V_{C_{z}}-\frac{Q_{C_{z}}}{C_{z}}$) are also listed in Table 4. The peak and lowest of different capacitor voltages of Figure 2, Figure 3 and Figure 4 follows the estimation method of Table 4, Table 5 and Table 6 respectively.

Table 7 shows the computation of the effective capacitance seen at the input nodes ($V_{a}$ and $V_{b}$) of CVMRs. The ripple and the curvature of the voltages of the input nodes of the CVMRs are determined by the corresponding equivalent capacitance values. The total stored energies of all the flying capacitors of 12P1OD-CVMR, 12P2OD-CVMR, and 12PDOD-CVMR at no load are $20.5CV_{p}^{2}$, $53CV_{p}^{2}$, $253CV_{p}^{2}$, respectively, when $V_{1o}=V_{1e}=V_{p}$. The amount of average stored energy of the flying capacitors reduces with the loading. The size of a capacitor is related to the energy stored, which is proportional to its bias voltage. Hence, the stored energy affects the overall size of the circuit. It can be observed that for the same amount of processed energy, the amount of stored energy is highest for 12PDOD-CVMR and lowest for 12P1OD-CVMR, with 12P2OD-CVMR having an intermediate value.

As can be seen in Table 4, the peak voltage of a capacitor is composed of an ideal voltage (when Q=0) and a voltage drop depends on the amount of charge processed by the previous lower indexed capacitors. This voltage drop is fundamental to the converter operation that depends on the converter topology, capacitance, and load. Using the amounts of charge the capacitors process, their voltage ripple, and thus, the capacitor’s lowest voltage can be derived.

It is also important to notice that the impact of stacking more capacitors in series, as in the 12P1OD-CVMR configuration, results in larger voltage drops from the ideal values, while the voltage drops are significantly smaller in the 12PDOD-CVMR configuration. Larger voltage drops lead to a reduction in the overall voltage gain of the converter. Therefore, one could favor 12PDOD-CVMR over 12P1OD-CVMR. However, on the other hand, while the operating voltage for 12P1OD-CVMR capacitors is limited to 2 kV for 12P1OD-CVMR, the voltages for 12PDOD-CVMR capacitors increase linearly close to the output voltage $V_{o}$ for higher index capacitors. The high operating voltages lead to a limited selection of capacitors and ones with large sizes and low capacitance density. 12P2OD-CVMR has a compromise with lower voltage requirements compared with 12PDOD-CVMR and lower voltage drops compared with 12P1OD-CVMR. It has to be noted that if MOSFETs are used in place of diodes of CVMRs, and all the even or odd indexed MOSFETs were turned on together, their drain and source terminals would have significantly different potentials at the turn-on instant, which would have resulted in current spikes and hard-charging/discharging loss for the flying capacitors. However, when diodes are used, the actual operation of the CVMRs is way more complicated and beyond the scope of this work. All the even or odd indexed diodes do not conduct together [29,30]. A diode starts conducting only when its cathode potential becomes equal to the anode potential. Hence, no current spike and complete soft charge/discharge [31] is ensured for the CVMRS. The actual operation does not change the capacitor voltages much; it changes the RMS current magnitudes even though the charge flow remains the same.

### 2.2. HGSME Families

The CVMRs require an AC voltage between their two input nodes $N_{a}$ and $N_{b}$. Based on the generation of this AC voltage and the shape of the AC voltage, different families of converters have been proposed, listed below for converter families.

**Figure 5:**
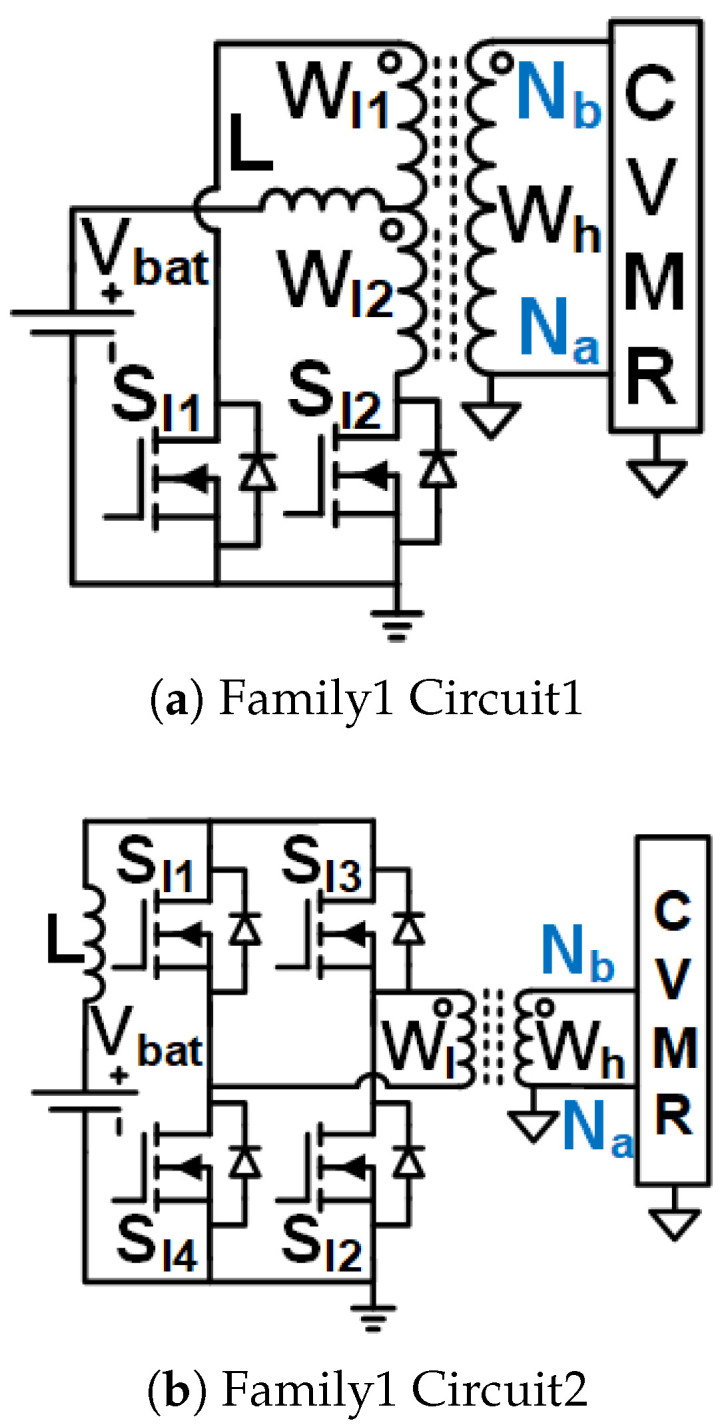
UHGH converters of Family1.

**Figure 6:**
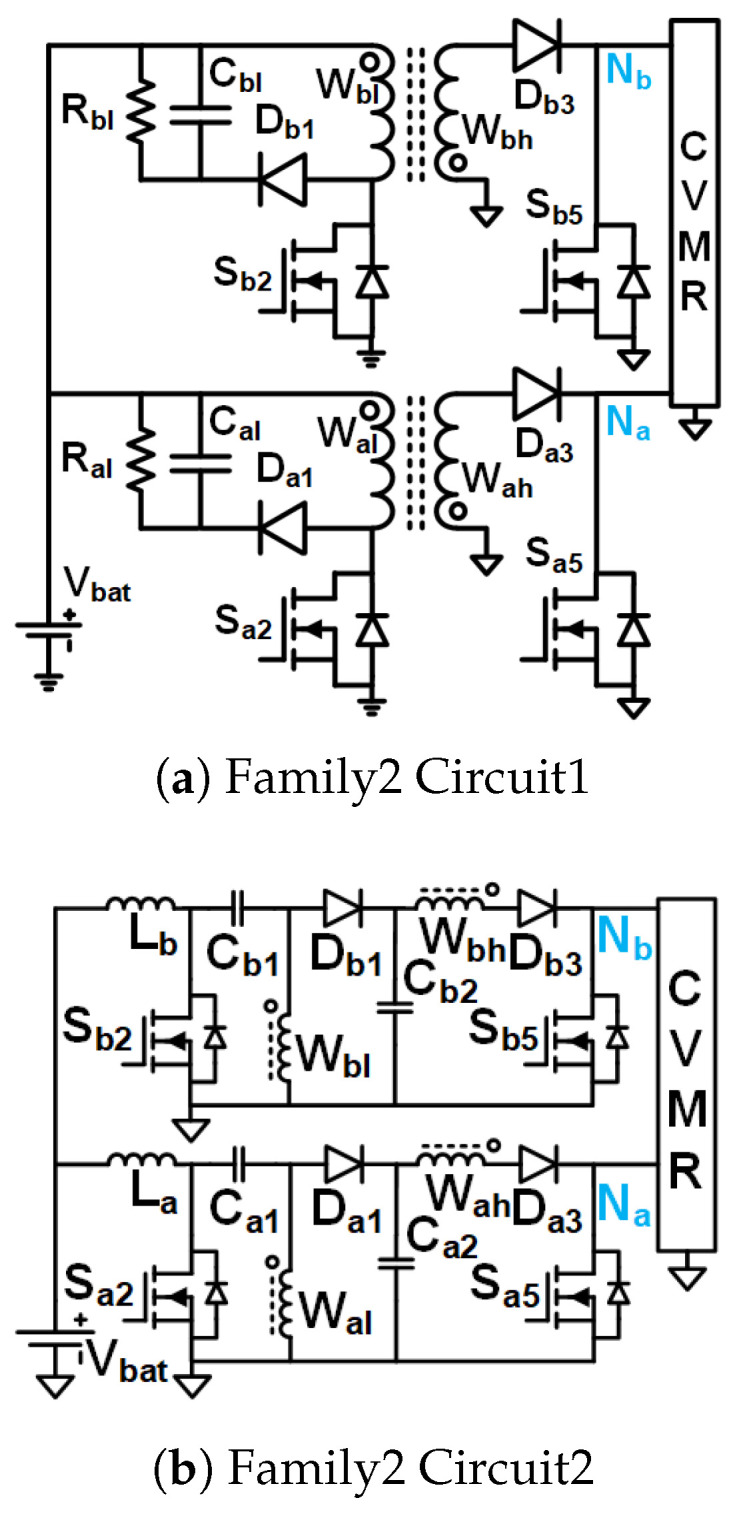
UHGH converters of Family2.

**Figure 7:**
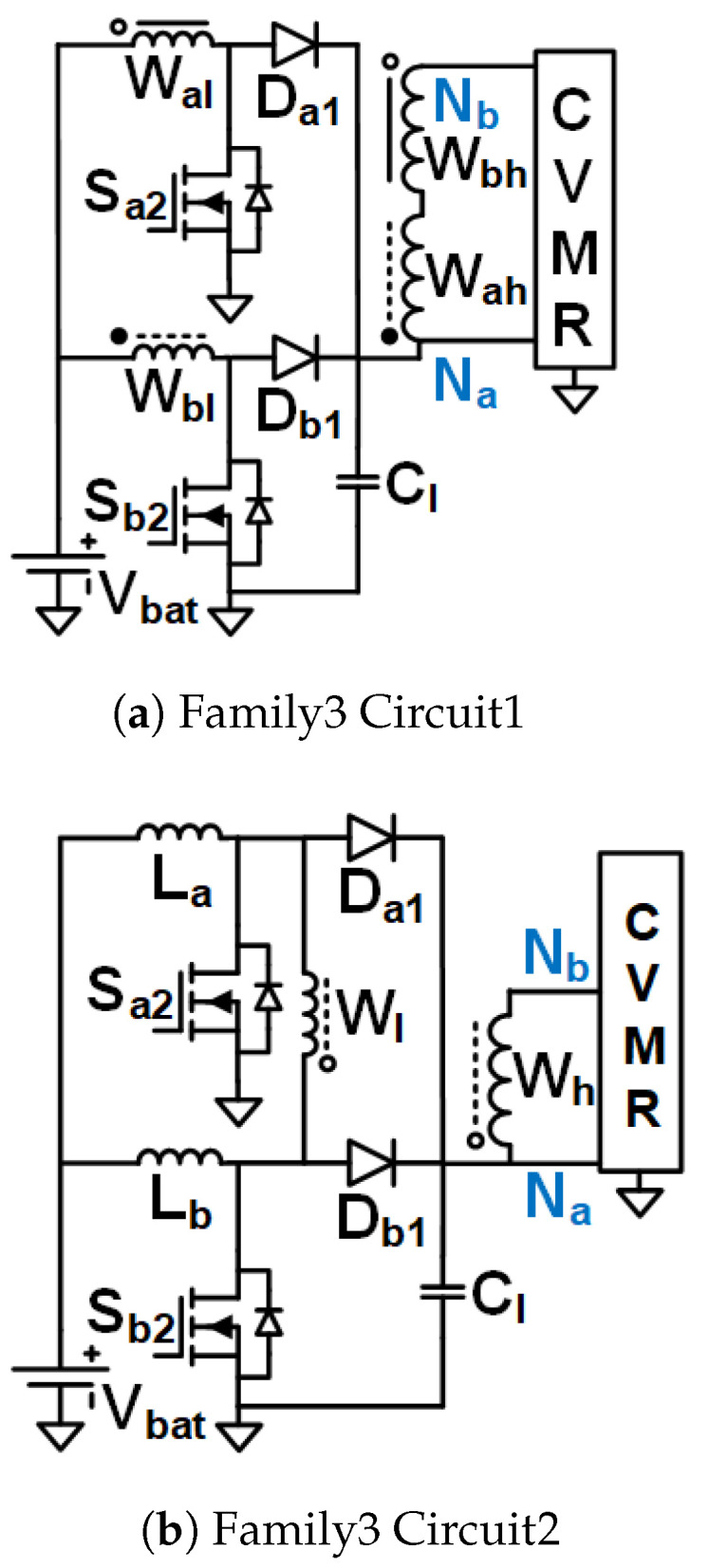
UHGH converters of Family3.

**Figure 8:**
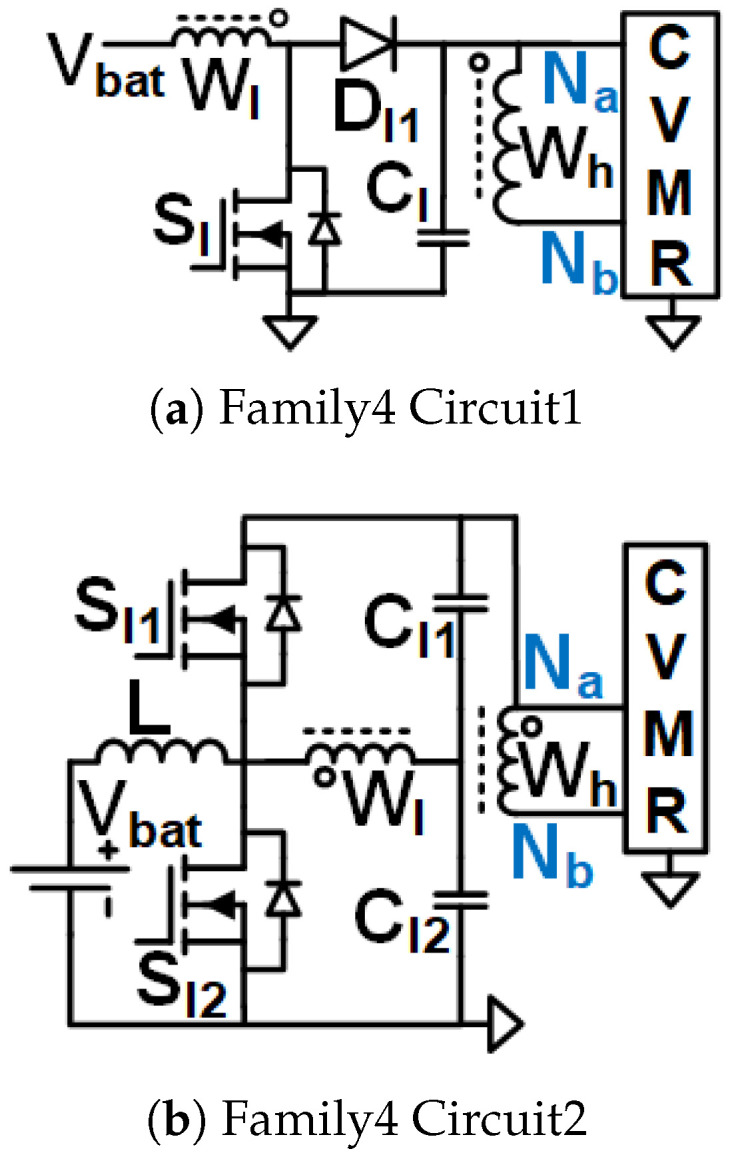
UHGH converters of Family4.

1. Family1 consists of isolated symmetrical non-interleaved converters, shown in Figure 5. These converters use transformers (no energy storage in the core). They have an inductor right next to the input. As the inductor current can not break, the switches need to be operated at duty cycles greater than 0.5. Boost-type voltage gain is achieved with the duty cycle. They require two gate pulses 180° phase-shifted from each other. They result in the symmetrical operation of the CVMR charging and discharging phases.
2. Family2 consists of two interleaved Current Pulse Generators (PCSos) using coupled inductors (stores energy in the core), shown in Figure 6. They require two gate pulses 180° phase-shifted from each other. They result in the symmetrical operation of the CVMR charging and discharging phases. The physical operation of the phase-A circuit is completely independent of the operation of the circuit of phase-B.
3. Family3 consists of two interleaved dependent structures with stacked output capacitors, shown in Figure 7. They require two gate pulses 180° phase-shifted from each other. They result in the symmetrical operation of the CVMR charging and discharging phases. The physical operation of the phase-A circuit is interdependent on the operation of the circuit of phase-B. They have stacked output capacitors at the output.
4. Family4 consists of converters with one gating pulse and stacked output capacitors, shown in Figure 8. This family results in the asymmetrical operation of the CVMR charging and discharging phases.

## 3. Operation of the UHGHs

### 3.1. Family1 Circuit1

**Figure 9:**
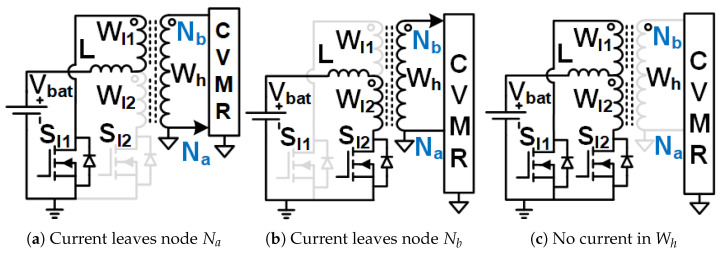
Family1 Circuit1 modes.

**Figure 10:**
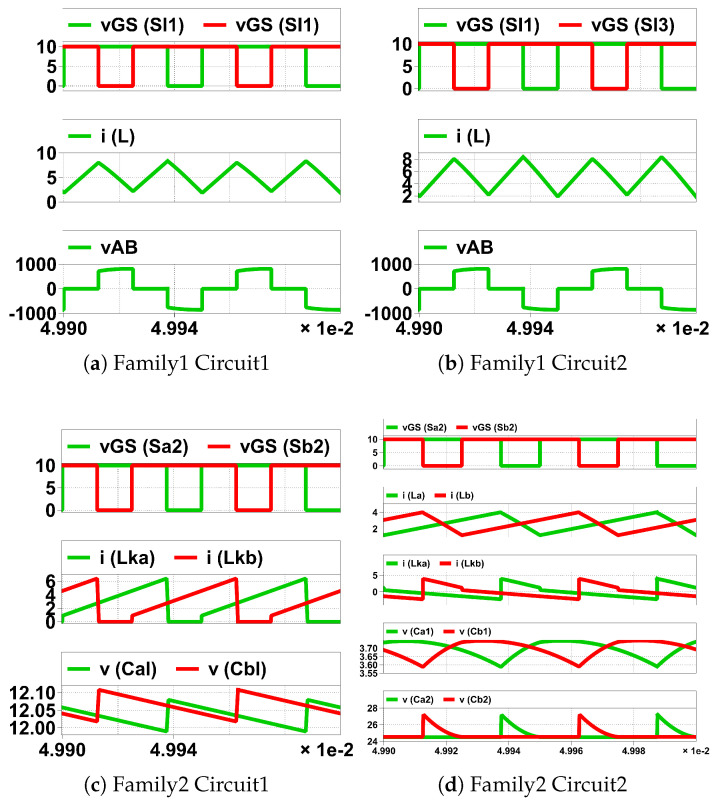

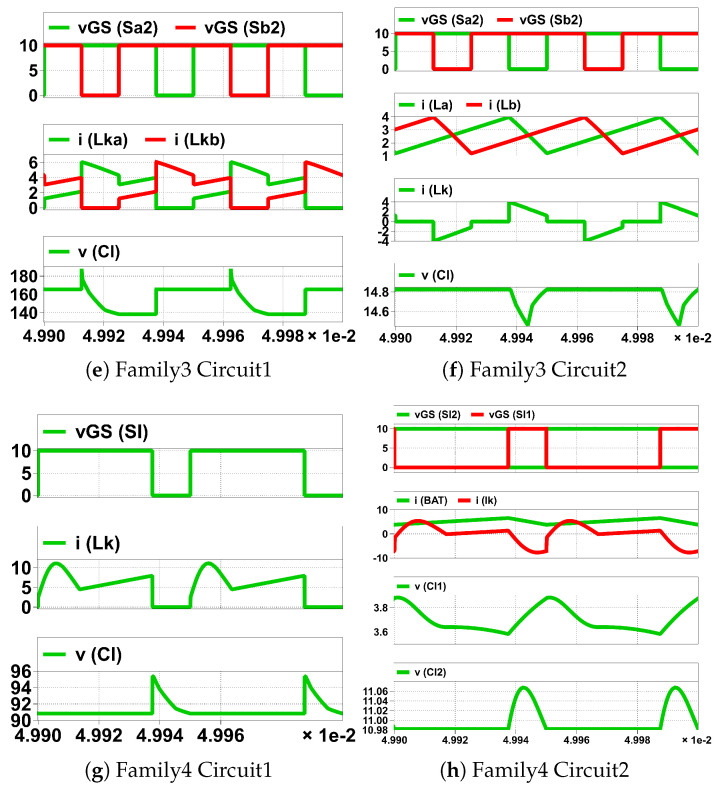
Simulation of HGSME of the UHGH converters. In horizontal scale ×1e-2 means $\times 10^{-2}$, unit is Second.

The gate pulses of $S_{l1}$ and $S_{l2}$ have an equal duty cycle higher than 50%, but they are 180° phase-shifted from each other. CCM operation with nearly constant current through the inductor *L* is assumed. As shown in Figure 9a,b when $S_{l1}$ ($S_{l2}$) is on, $S_{l2}$ ($S_{l1}$) is off, the current of inductor *L* passes through $W_{l1}$ ($W_{l2}$), current enters $N_{b}$ ($N_{a}$), leaves $N_{a}$ ($N_{b}$) of HGSME to achieve the MMF balance of the transformer. As shown in Figure 9c When $S_{l1}$ and $S_{l2}$ are both on, the current of inductor *L* is equally divided into two paths, one through $W_{l1}$ and $S_{l1}$ and the other through $W_{l2}$ and $S_{l2}$, no current is seen in $W_{h}$ because the MMF balance of the transformer is already achieved by the currents through $W_{l1}$ and $W_{l2}$. Hence, the current through $W_{h}$ has negative and zero. Hence, it is a quasi-square waveform whose magnitude is a stepped-down version of the current through *L* by a factor of turn ratio of transformer $W_{1}$. The voltage of the magnetically coupled windings $W_{l1}$, $W_{l2}$) are quasi-square waves whose average is zero due to the volt-second balance of the windings and whose positive and negative peaks are decided by the volt second balance of *L*. The voltage waveform of $W_{h}$ is a stepped-up version of the voltage waveforms of $W_{l1}$ and $W_{l2}$ by a factor of turn ratio $W_{1}$ of the transformer. When off, the voltage across $S_{l1}$ ($S_{l2}$) is twice the voltage of $W_{l2}$ ($W_{l1}$). The quasi-square voltage and current waveforms of $W_{h}$ serve as the input to the CVMR. The core flux traverses all four quadrants of the B-H curve, and automatic core resetting happens in every switching period. Waveforms of Figure 10a validate the operation of HGSME of Family1 Circuit1.

### 3.2. Family1 Circuit2

**Figure 11:**
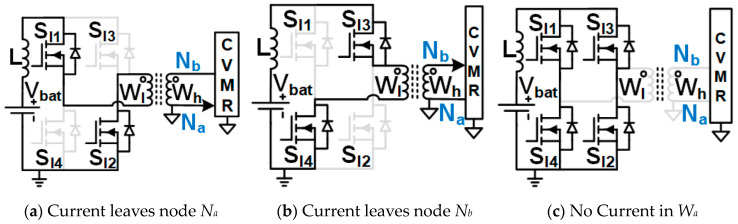
Family1 Circuit2 modes.

The gate pulses of $S_{l1}$ and $S_{l2}$ are identical. The gate pulses of $S_{l3}$ and $S_{l4}$ are identical. The gate pulses of $S_{l1}$ and $S_{l3}$ have an equal duty cycle higher than 50%, but they are 180° phase-shifted from each other. CCM operation with nearly constant current through the inductor *L*. As shown in Figure 11a,b when $S_{l1}$ ($S_{l3}$) is on, $S_{l3}$ ($S_{l1}$) is off, the current of inductor *L* leaves (enters) through the dot of $W_{l}$, current enters $N_{b}$ ($N_{a}$), leaves $N_{a}$ ($N_{b}$) of HGSME to achieve the MMF balance of the transformer. As shown in Figure 9c when $S_{l1}$ and $S_{l3}$ are both on, the current of inductor *L* is equally divided into two paths, one through $S_{l1}$ and $S_{l4}$ and the other through $S_{l3}$ and $S_{l2}$, no current is seen in $W_{l}$ and $W_{h}$. Hence, the current through $W_{h}$ has negative and zero. Hence, it is a quasi-square waveform whose magnitude is a stepped-down version of the current through *L* by a factor of turn ratio of transformer $W_{1}$. The voltage of $W_{l1}$ is quasi-square waves whose average is zero due to the volt-second balance of the winding and whose positive and negative peaks are decided by the volt-second balance of *L*. The voltage waveform of $W_{h}$ is a stepped-up version of the voltage waveform of $W_{l}$ by a factor of the turn ratio $W_{1}$ of the transformer. When off, the voltage across $S_{l1}$ ($S_{l3}$) is the voltage of $W_{l}$. The quasi-square voltage and current waveforms of $W_{h}$ serve as the input to the CVMR. The core flux traverses all four quadrants of the B-H curve, and automatic core resetting happens in every switching period. Waveforms of Figure 10b validate the operation of HGSME of Family1 Circuit2.

### 3.3. Family2 Circuit1

**Figure 12:**
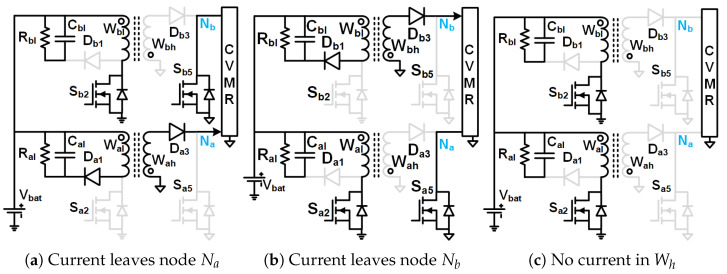
Family2 Circuit1 modes.

The gate pulses of $S_{a2}$ and $S_{b2}$ have an equal duty cycle higher than 50%, but they are 180° phase-shifted from each other. CCM operation is assumed. As shown in Figure 12b when $S_{a2}$ is on and $S_{b2}$ is off, $D_{a3}$ and $S_{b5}$ is off, $D_{b3}$ and $S_{a5}$ is on, current leaves $N_{b}$ and enters $N_{a}$ of HGSME, the current of magnetizing inductance of $W_{a}$ increases linearly and $W_{b}$ decreases linearly. As shown in Figure 12a when $S_{b2}$ is on and $S_{a2}$ is off, $D_{b3}$ and $S_{a5}$ is off, $D_{a3}$ and $S_{b5}$ is on, current leaves $N_{a}$ and enters $N_{b}$ of HGSME, the current of magnetizing inductance of $W_{b}$ increases linearly and $W_{a}$ decreases linearly. As shown in Figure 12c when both $S_{a2}$ and $S_{b2}$ are on, $D_{a3}$, $S_{b5}$, $D_{b3}$ and $S_{a5}$ are off, no current leaves/enters $N_{a}$ and $N_{b}$, the current of magnetizing inductance of $W_{a}$ and $W_{b}$ both increases. Hence, the current output of HGSME has negative and zero. Hence, it is a quasi-square waveform. The voltage between $N_{a}$ and $N_{b}$ is a quasi-square wave whose positive and negative peaks are decided by the volt-second balance of the magnetizing inductances of $W_{a}$ and $W_{b}$. The voltage waveform of $W_{h}$ having zero average due to volt-second balance is a stepped-up version of the voltage waveform of $W_{l}$ by a factor of the turn ratio $W_{1}$ of the transformer. When $S_{a2}$ ($S_{b2}$) is off, the voltage across it is $V_{bat}+\frac{V_{a}}{W_{1}}$. When $D_{a3}$ is off and $S_{a5}$ is on ($D_{b3}$ is off and $S_{b5}$), the voltage across it is $W_{1}V_{bat}$.The quasi-square voltage and current waveforms generated by HGSME serve as the input to the CVMR. The core flux of $W_{a}$ and $W_{b}$ stays in the first quadrant of the B-H curve. Waveforms of Figure 10c validate the operation of HGSME of Family2 Circuit1.

### 3.4. Family2 Circuit2

**Figure 13:**
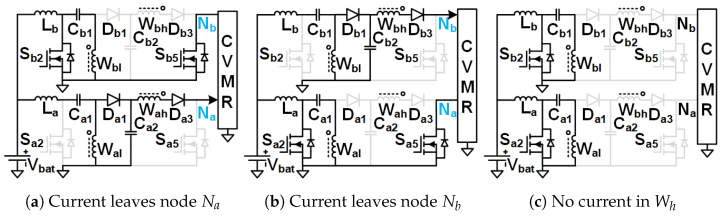
Family2 Circuit2 modes.

The gate pulses of $S_{a2}$ and $S_{b2}$ have an equal duty cycle higher than 50%, but they are 180° phase-shifted from each other. CCM operation is assumed for $L_{a}$ and $L_{b}$. As shown in Figure 13b when $S_{a2}$ is on and $S_{b2}$ is off, $D_{a1}$, $D_{a3}$ and $S_{b5}$ is off, $D_{b1}$, $D_{b3}$ and $S_{a5}$ is on, current leaves $N_{b}$ and enters $N_{a}$ of HGSME, the current of magnetizing inductance of $W_{a}$ increases linearly and $W_{b}$ decreases linearly, voltage across $W_{al}$ is $-V_{C_{a1}}$ and voltage across $W_{bl}$ is $V_{C_{b2}}$. As shown in Figure 13a When $S_{b2}$ is on and $S_{a2}$ is off, $D_{b1}$, $D_{b3}$ and $S_{a5}$ is off, $D_{a1}$, $D_{a3}$ and $S_{b5}$ is on, current leaves $N_{a}$ and enters $N_{b}$ of HGSME, the current of magnetizing inductance of $W_{b}$ increases linearly, and $W_{a}$ decreases linearly, voltage across $W_{al}$ is $V_{C_{a2}}$ and voltage across $W_{bl}$ is $-V_{C_{b1}}$. As shown in Figure 13c when both $S_{a2}$ and $S_{b2}$ are on, $D_{a1}$, $D_{a3}$, $S_{b5}$, $D_{b1}$, $D_{b3}$ and $S_{a5}$ are off, no current leaves/enters $N_{a}$ and $N_{b}$, the current of magnetizing inductance of $W_{a}$ and $W_{b}$ both increases, voltage across $W_{al}$ is $-V_{C_{a1}}$ and voltage across $W_{bl}$ is $-V_{C_{b1}}$. Hence, the current output of HGSME has positive, negative, and zero. Hence, it is a quasi-square waveform. The voltage between $N_{a}$ and $N_{b}$ is a quasi-square wave whose positive and negative peaks are decided by the volt-second balance of the magnetizing inductances of $W_{h}$. The voltage waveform of $W_{h}$ having zero average due to volt-second balance is a stepped-up version of the voltage waveform of $W_{l}$ by a factor of the turn ratio $W_{1}$ of the transformer. The voltage rating of $S_{a2}$, $D_{a1}$, $S_{b2}$, $D_{b1}$ is $V_{C_{a1}}+V_{C_{a2}}$. The voltage rating of $D_{a3}$ and $D_{b3}$ is $W_{1}V_{C_{a1}}-V_{C_{a2}}$. The voltage rating of $S_{a5}$ and $S_{b5}$ is $W_{1}V_{C_{a1}}+V_{C_{a2}}$. The quasi-square voltage and current waveforms generated by HGSME serve as the input to the CVMR. The core flux of $W_{a}$ and $W_{b}$ stays in the first quadrant of the B-H curve. Waveforms of Figure 10d validate the operation of HGSME of Family2 Circuit2.

### 3.5. Family3 Circuit1

**Figure 14:**
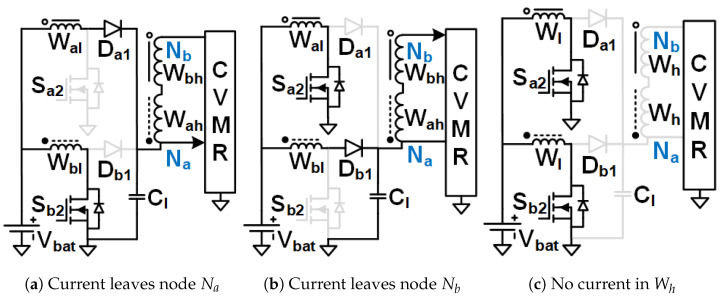
Family3 Circuit1 modes.

The gate pulses of $S_{a2}$ and $S_{b2}$ have an equal duty cycle, but they are 180° phase-shifted from each other. CCM operation is assumed for the magnetizing currents of $W_{al}$ and $W_{bl}$. As shown in Figure 14b when $S_{a2}$ is on and $S_{b2}$ is off, $D_{a1}$ and is off, $D_{b1}$ is on, current leaves $N_{b}$ and enters $N_{a}$ of HGSME, the magnetizing current of $W_{al}$ increases linearly and $L_{bl}$ decreases linearly. As shown in Figure 14a when $S_{b2}$ is on and $S_{a2}$ is off, $D_{b1}$ is off, $D_{a1}$ is on, current leaves $N_{a}$ and enters $N_{b}$ of HGSME, the magnetizing current of $W_{bl}$ increases linearly and $W_{al}$ decreases linearly. As shown in Figure 14c when both $S_{a2}$ and $S_{b2}$ are on for D≥0.5, $D_{a1}$ and $D_{b1}$ are off, zero voltage and current out of HGSME because the induced voltages of $W_{ah}$ and $W_{bh}$ cancel each other, the magnetizing current of $W_{al}$ and $W_{bl}$ both increases. When both $S_{a2}$ and $S_{b2}$ are off for D≤0.5, $D_{a1}$ and $D_{b1}$ are on, zero voltage and current out of HGSME, because the induced voltages of $W_{ah}$ and $W_{bh}$ cancel each other, the magnetizing current of $W_{al}$ and $W_{bl}$ both decreases. The voltage waveform of $W_{h}$ has zero average due to volt-second balance and has a quasi-square wave. When off, the voltage across $S_{a2}$, $D_{a1}$, $S_{b2}$, $D_{b1}$ is $V_{C_{l}}$. The quasi-square voltage waveforms generated by HGSME serve as the input to the CVMR. The core flux of the transformer traverses all four quadrants of the B-H curve. Waveforms of Figure 10e validate the operation of HGSME of Family3 Circuit1.

### 3.6. Family3 Circuit2

**Figure 15:**
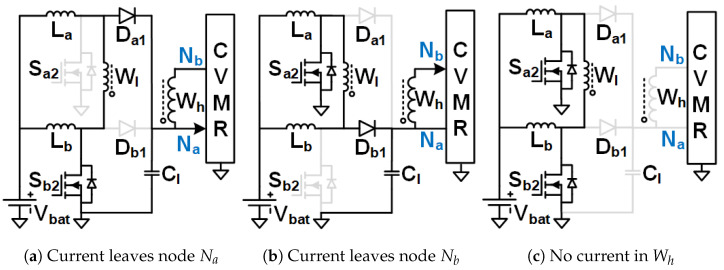
Family3 Circuit2 modes.

The gate pulses of $S_{a2}$ and $S_{b2}$ have an equal duty cycle, but they are 180° phase-shifted from each other. CCM operation is assumed for $L_{a}$ and $L_{b}$. As ahown in Figure 15a when $S_{a2}$ is on and $S_{b2}$ is off, $D_{a1}$ and is off, $D_{b1}$ is on, current leaves $N_{a}$ and enters $N_{b}$ of HGSME, the current of $L_{a}$ increases linearly and $L_{b}$ decreases linearly. As shown in Figure 15b When $S_{b2}$ is on and $S_{a2}$ is off, $D_{b1}$ is off, $D_{a1}$ is on, current leaves $N_{b}$ and enters $N_{a}$ of HGSME, the current of $L_{b}$ increases linearly and $L_{a}$ decreases linearly. As shown in Figure 15c when both $S_{a2}$ and $S_{b2}$ are on for D≥0.5, $D_{a1}$ and $D_{b1}$ are off, zero voltage and current through $W_{l}$ and $W_{h}$, the current of $L_{a}$ and $L_{b}$ both increases. When both $S_{a2}$ and $S_{b2}$ are off for D≤0.5, $D_{a1}$ and $D_{b1}$ are on, zero voltage and current through $W_{l}$ and $W_{h}$, the current of $L_{a}$ and $L_{b}$ both increases. Hence, the current output of HGSME has negative and zero. Hence, it is a quasi-square waveform. The voltage across $W_{l}$ quasi-square wave whose positive and negative peaks are $V_{C_{l}}$. The voltage waveform of $W_{h}$ having zero average due to volt-second balance is a stepped-up version of the voltage waveform of $W_{l}$ by a factor of the turn ratio $W_{1}$ of the transformer. When off, the voltage across $S_{a2}$, $D_{a1}$, $S_{b2}$, $D_{b1}$ is $V_{C_{l}}$. The quasi-square voltage and current waveforms generated by HGSME serve as the input to the CVMR. The core flux of the transformer traverses all four quadrants of the B-H curve. Waveforms of Figure 10f validate the operation of HGSME of Family3 Circuit2.

### 3.7. Family4 Circuit1

**Figure 16:**
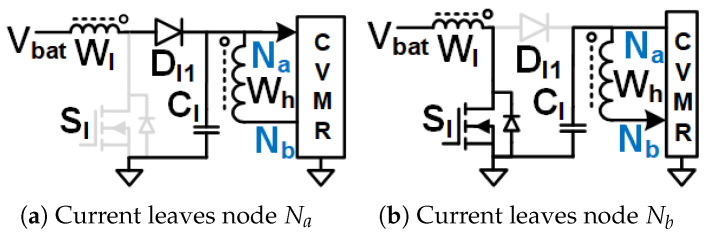
Family4 Circuit1 modes.

Assuming CCM operation of the magnetizing inductance of $W_{l}$, $C_{C_{l}}$ is obtained through the volt-second balance of magnetizing inductance off $W_{l}$. As shown in Figure 16b when $S_{l}$ is on, the magnetizing current of $W_{l}$ increases linearly, and the voltage of $W_{l}$ is $V_{bat}$, the current leaves $N_{b}$ and enters $N_{a}$ of HGSME. As shown in Figure 16a when $S_{l1}$ is off, the magnetizing current of $W_{l}$ decreases linearly, voltage of $W_{l}$ is $-V_{C_{l}}$, the current leaves $N_{a}$ and enters $N_{b}$ of HGSME. The voltage across $W_{l}$ has zero average. The voltage across $W_{h}$ is a stepped-up version of the voltage across $W_{l}$. Hence, the voltage of $W_{h}$ has negative and positive polarities. The voltage waveform generated by HGSME serves as the CVMR input. The core flux of the transformer stays in the first quadrant of the B-H curve. Waveforms of Figure 10g validate the operation of HGSME of Family4 Circuit1.

### 3.8. Family4 Circuit2

**Figure 17:**
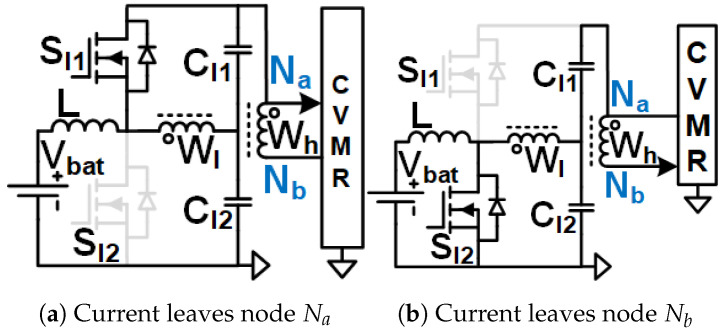
Family4 Circuit2 modes.

The gate pulses of $S_{l2}$ and $S_{l1}$ are complementary to each other. CCM operation is assumed for *L*. As shown in Figure 17b when $S_{l2}$ is on, and $S_{l1}$ is off, the current of *L* increases linearly, the current leaves dot of $W_{l}$, the current leaves $N_{b}$ and enters $N_{a}$ of HGSME, the voltage of $W_{l}$ is $V_{C_{l2}}$. As shown in Figure 17a when $S_{l1}$ is on, and $S_{l2}$ is off, the current of *L* decreases linearly, the current enters dot of $W_{l}$, the current leaves $N_{a}$ and enters $N_{b}$ of HGSME, voltage of $W_{l}$ is $-V_{C_{l1}}$. The voltage of $W_{h}$ is a stepped-up version of the voltage across $W_{l}$. Hence, the current and voltage of $W_{h}$ both have negative and positive polarities. The voltage and current waveforms generated by HGSME serve as the CVMR input. The core flux of the transformer stays in the first quadrant of the B-H curve. Waveforms of Figure 10h validate the operation of HGSME of Family4 Circuit2.

Table 8 contains an approximate theoretical analysis of the UHGH converters. Table 9 contains simulation results of the HGSME of the UHGH converters.

## 4. Validation of the UHGHs

### 4.1. Simulation of the UHGHs

**Figure 18:**
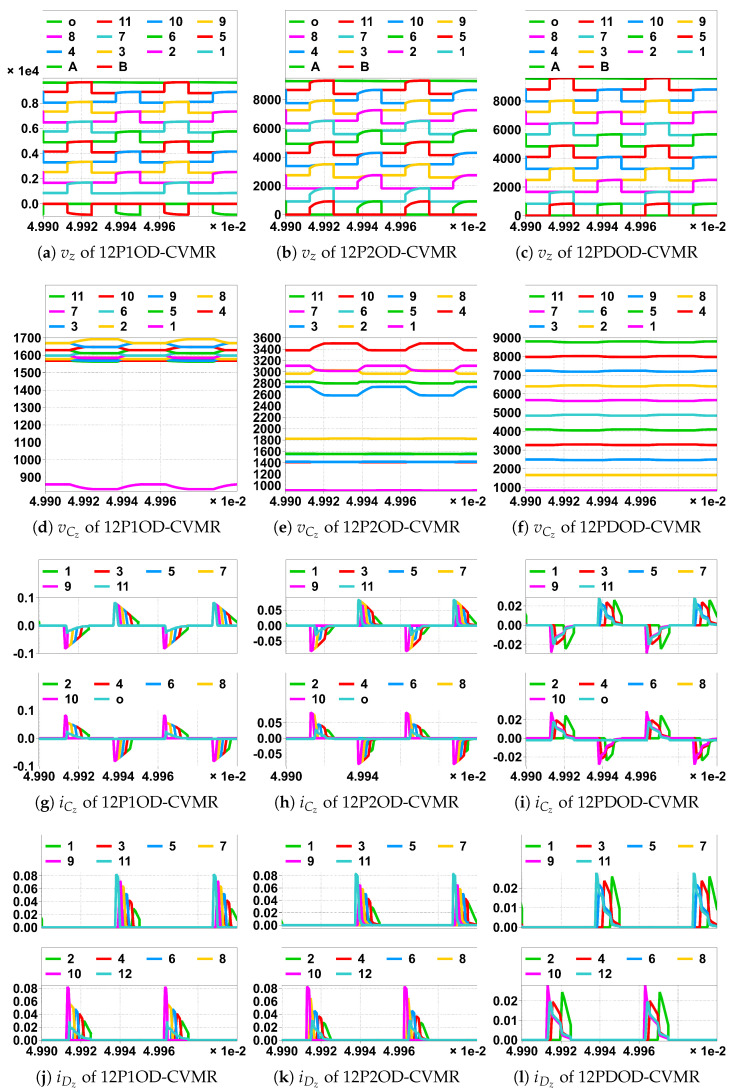
Simulation of CVMR part of the UHGH converters of Family2; (legend number is the value of *z*). In horizontal scale ×1e-2 means $\times 10^{-2}$, unit is Second.

**Figure 19:**
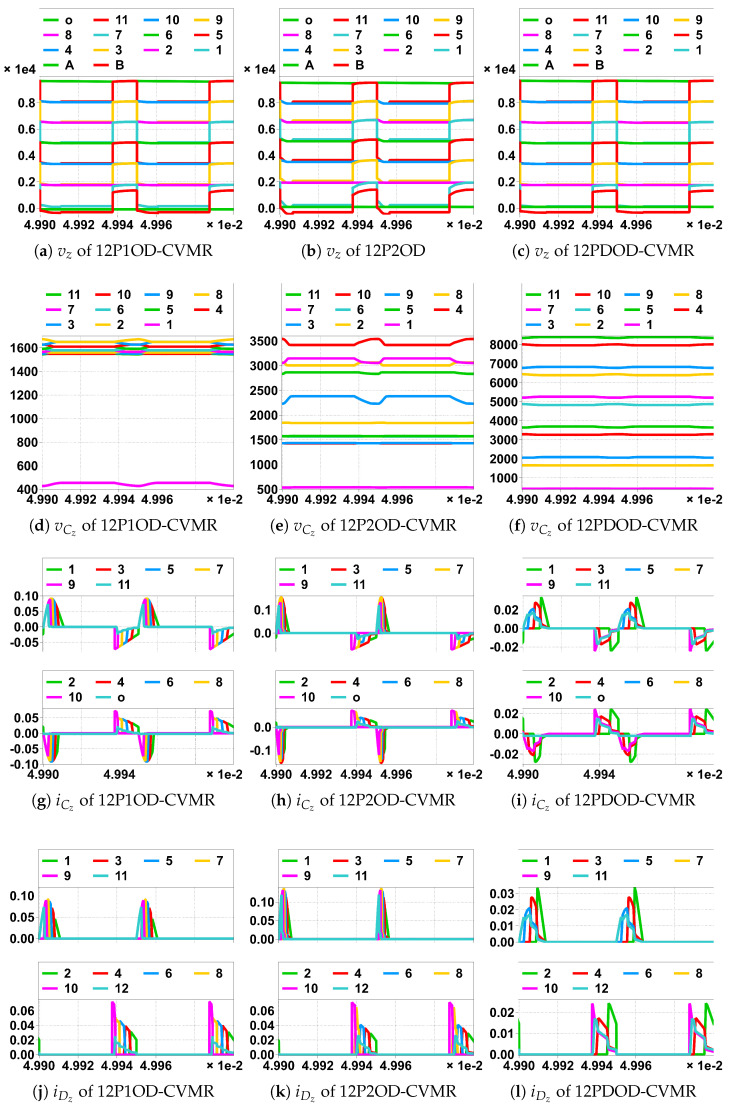
Simulation of CVMR part of the UHGH converters of Family4; (legend number is the value of *z*). In horizontal scale ×1e-2 means $\times 10^{-2}$, unit is Second.

For Family2, both $N_{a}$ and $N_{b}$ are high voltage pulses with respect to the ground. Hence, all the even as well as the odd indexed nodes of the CVMR attain peak and lowest voltage levels periodically, which can be seen in Figure 18a–c. However, in Family4, $N_{a}$ is at a fixed voltage with respect to the ground. Hence, only the odd indexed nodes of the CVMR attain peak and lowest voltage levels periodically, but all the even indexed nodes stay at fixed voltage with respect to ground, which can be seen in Figure 19a–c. $v_{ab}$ is symmetric for Family2 seen from Figure 18g–i. $v_{ab}$ is asymmetric for Family4 seen from Figure 19g–i.

The peak and lowest of different capacitor voltages of Figure 18d and Figure 19d follows the estimation method of Table 4. The peak and lowest of different capacitor voltages of Figure 18e and Figure 19e follows the estimation method of Table 5. The peak and lowest of different capacitor voltages of Figure 18f and Figure 19f follows the estimation method of Table 6. Subtracting the two consecutive diode currents of Figure 18j–l (Figure 19j–l), gives the corresponding capacitor currents of Figure 18g–i (Figure 19g–i), respectively. It has been observed that any higher-indexed diode starts conducting before any lower-indexed diode for 12P1OD-CVMR.

### 4.2. Experiment of a UHGH

**Figure 20:**
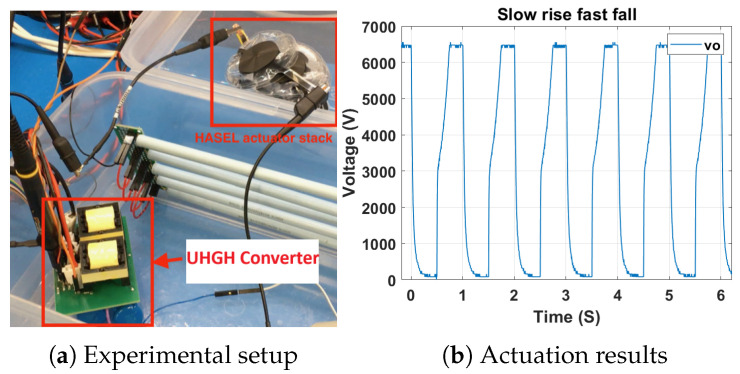
Demonstration of a UHGH converter with HASEL.

An experiment with the UHGH converter with HGSME of Family2 Circuit1 and 12PDOD-CVMR is presented here. Figure 20a shows the experimental setup. It achieves significantly improved performance to [32]. The composition of the circuit is listed in Table 10. Figure 20b shows the practical demonstration of a UHGH converter with HASEL. In total, 11 HASEL electrostatic actuators, which are black color disc-shaped objects, are electrically connected in parallel. The green color board is the UHGH converter. A DC power supply, which emulates the battery, has been used to supply a fixed voltage of $V_{bat}=3.7$ V at the converter input. The actuation process is equivalent to the charging and discharging of an RC network. Actuators can be modeled as capacitive loads. Load resistance of 5 MΩ value is connected in parallel to the actuator stack to discharge them. The rate of fall of the output voltage is small due to the high value of load resistance. The rate of rise of the output voltage is high due to the low equivalent output resistance of the converter. The experiments demonstrated ∼7 kV output from 3.7 V input, max. The output power was 25 W at a power density of more than 10 W/in^2^ (considering the summation of the volume of all components) and a weight of less than 170 g. Comparison with a commercial high voltage power electronic driver circuit [33] is presented in Table 11.

TMS320F28379D microcontroller generates the pulses using the EPWM module. The Automated Duty Cycle and Frequency Control is performed by keeping the input voltage fixed and periodically repeating the following sequence: First, the duty cycle jumps from 0 to 0.5, then gradually increases from 0.5 to 0.75 at a controllable rate. The actuator stack charges through the converter and expands. Then, the duty cycle is kept fixed at 0.75 for some time according to mechanical actuation frequency. After that, the duty cycle is made zero. The actuator stack discharges through the load resistance and contracts. Lodh and Le [34] present a similar demonstration with negative CVMR with Figure 6a. A detailed demonstration of Figure 7a can be found in [35].

## 5. Discussions

### 5.1. Advantages of the UHGH Converters

The operations of the UHGH converters containing CVMR and HGSME reveal several key advantages that can be classified into the following broad categories:

1. Smaller Size and Weight: The gain (∼10×) provided by the CVMR significantly reduces the voltage gain required from the coupled inductor stage, allowing the turn ratio of coupled inductors to be small. This is beneficial in two ways. First, the number of winding turns and associated intra-winding insulation of the high-voltage winding is reduced, leading to smaller and lighter coupled inductors (both bobbins and cores). Second, the output of the coupled inductors only needs to provide a small fraction of the total output voltage. Hence, the voltage stress, and insulation are reduced further, resulting in a further reduction in size and weight. Smaller volumes and weights for the entire coupled inductors are critical for the mobility and efficiency of soft robots. Almost all the UHGH topologies (except the one in Figure 8a) either employ an interleaved operation or contain an inductor right next to the input voltage source. Hence, the input current ripple is small, which enables a smaller and more compact input filter design.
2. Low Cost: Even though the output voltage is very high, the UHGH converter enables the use of standard, compact, low-voltage semiconductor devices (switches and diodes) that are readily available off the shelf. This makes the solution low-cost and easily mass-producible.
3. Adjustable Output: Efficient fine output voltage adjustment can be achieved with simple duty cycle modulation. This makes the UHGH converters usable for actuators with a large voltage range of 4–12 kV as well as lower.
4. Scalability and Flexibility: On one hand, CVMR can easily be reconfigured with different numbers of capacitors, diodes, and levels, which results in different conversion ratios. On the other hand, the coupled inductor stage can have different turn ratios optimal for specific applications. Hence, the UHGH converter topologies can easily be optimized for a wide range of applications with different voltage requirements without changing the basic architecture.
5. Reliability: All the UHGH converter topologies make sure that there is an alternate path for the flow of the leakage inductor current when one path is interrupted by turning off the switches. Hence, the possibility of damage to the converter due to the generation of excessively high overvoltage is eliminated. This ensures reliable operation.
6. Low Loss: One of the major loss components is the loss associated with the high voltage winding of the coupled inductors or transformers due to the presence of parasitic interwinding and intra-winding capacitances. The smaller winding structure of the UHGH converters results ensures the small value of those parasitic capacitances. Hence, the loss associated with periodically charging and discharging those parasitic capacitors is less in the UHGH converters.
7. Reduction of Ripple and Noise: The reduction in parasitic winding capacitances reduces unwanted spikes in the winding current. This reduces electromagnetic interference (EMI) noise and increases battery life.

### 5.2. Comparison of the UHGH Converters

1. Interleaved Structure: To support large output power, the current in the low-voltage input side, where the 2–5 V Li-ion battery is used for untethered applications, can be very high. The interleaved operation employed in the UHGH converters of Figure 6 and Figure 7a,b enables current sharing to theoretically half copper $I^{2}R$ loss of the coupled inductor stage.
2. Symmetrical Operation of CVMR: Except for the two topologies Figure 8, all other UHGH converter topologies employ symmetrical operation of the CVMR part. The voltage ($V_{1o}$) between nodes $N_{a}$ and $N_{b}$ during the period of conduction of odd indexed diodes is the same as the voltage ($V_{1e}$) between nodes $N_{a}$ and $N_{b}$ during the period of conduction of even indexed diodes.
3. Continuous Input Current: The UHGH converter topologies of Figure 5, Figure 6b, Figure 7b and Figure 8b have uncoupled inductors, right next to the input. Hence, the input current is continuous and has a low ripple. Among these, the topologies of Figure 6b and Figure 7b have two interleaved inductors right next to the input. Hence, they have the least input current ripple. The remaining topologies have pulsating input currents. Among these, the topologies of Figure 6a have less input current ripple due to interleaved operation. The topology of Figure 8a is expected to have a high input current ripple.
4. Number of Components and Component Ratings: Even though the topology of Figure 6b has continuous input current with a small ripple, it requires a comparatively large number of components and a large value of the capacitors in the HGSME. Topologies of Figure 7a and Figure 8a need comparatively higher voltage rating switches that need to carry the high input current. Topologies of Figure 5 and Figure 8 require the highest turn ratio. Topologies of Figure 7a require a moderate turn ratio. Topologies of Figure 7b require the least turn ratio. The higher rating of the components causes higher loss, cost, and space.
5. Number of Levels of CVMRs: The CVMRs of Family2 can have either an even or an odd number of levels without change in CVMR input terminals because the voltage at both the nodes $N_{a}$ and $N_{b}$ is not fixed all the time and fluctuates symmetrically. On the other hand, the CVMRs of Family1, Family3, and Family4 either need to have an even number of levels because the voltage at the node $N_{a}$ is fixed and the voltage at the node $N_{b}$ is not fixed all the time, or the input terminals of the CVMRs need to swap to have the odd number of levels.

Table 12 contains descriptions and features of the UHGH converters. Table 13 contains a Comparison of HGSME Output Voltages and Flying Capacitor Voltages of the CVMR Part of the UHGH Converters.

## 6. Conclusions

In this work, eight topologies of UHGH converters are with the operation and validation results. Efficient synchronous operation between the HGSME and CVMR pair is the key to the UHGH converters. The converter analysis, design, and development show a promising solution for soft mobile robots that can support an extremely large conversion ratio of ∼2000× while achieving high efficiency, high output power, and adjustable output voltage in a compact size. Analyses are performed for the output voltage drop and the peak, median, and lowest voltage of all the flying capacitors of the three different CVMRs. A qualitative comparison between the UHGH converters is presented. A comparison of the high-gain converters and high output voltage converters is presented. The UHGH converters support much higher power than commercial products with similar voltage output and power density. Hence, the UHGH converters are suitable for a large stack of HASEL actuators requiring significant power. The best UHGH converter of Figure 5a has been demonstrated due to its low component count, absence of complicated high-side switch driver, isolation capability, current sharing (through interleaving) in the high current input side, symmetrical operation of CVMR, unchanged connection at the junction of HGSME and CVMR for both even and odd levels of CVMR, ready commercial availability of coupled inductor with high turn ratio. The experiments demonstrated ∼7 kV output from 3.7 V input, max. output power of 25 W at a power density of more than 10 W/in^2^, weight of less than 170 g.

## Figures and Tables

**Table 1:** KCL to relate different $Q_{C_{z}}$ in 12P(N)OD-CVMR.

N=1		N=2		N→∞	
**Curr. Enters ** $N_{a}$	**Curr. Enters ** $N_{b}$	**Curr. Enters ** $N_{a}$	**Curr. Enters ** $N_{b}$	**Curr. Enters ** $N_{a}$	**Curr. Enters ** $N_{b}$
	$Q_{C_{1}}=Q_{C_{2}}-Q$		$Q_{C_{1}}=Q_{C_{2}}$		$Q_{C_{1}}=Q_{C_{2}}$
$Q_{C_{2}}=Q_{C_{3}}$	$Q_{C_{3}}=Q_{C_{4}}-Q$	$Q_{C_{2}}=Q_{C_{3}}-Q_{C_{4}}$	$Q_{C_{3}}=Q_{C_{4}}+Q_{C_{1}}$	$Q_{C_{2}}=Q_{C_{3}}$	$Q_{C_{3}}=Q_{C_{4}}$
$Q_{C_{4}}=Q_{C_{5}}$	$Q_{C_{5}}=Q_{C_{6}}-Q$	$Q_{C_{4}}=Q_{C_{7}}+Q_{C_{5}}$	$Q_{C_{5}}=Q_{C_{6}}$	$Q_{C_{4}}=Q_{C_{5}}$	$Q_{C_{5}}=Q_{C_{6}}$
$Q_{C_{6}}=Q_{C_{7}}$	$Q_{C_{7}}=Q_{C_{8}}-Q$	$Q_{C_{6}}=Q_{C_{7}}-Q_{C_{8}}$	$Q_{C_{7}}=Q_{C_{8}}+Q$	$Q_{C_{6}}=Q_{C_{7}}$	$Q_{C_{7}}=Q_{C_{8}}$
$Q_{C_{8}}=Q_{C_{9}}$	$Q_{C_{9}}=Q_{C_{10}}-Q$	$Q_{C_{8}}=Q_{C_{11}}+Q_{C_{9}}$	$Q_{C_{9}}=Q_{C_{10}}$	$Q_{C_{8}}=Q_{C_{9}}$	$Q_{C_{9}}=Q_{C_{10}}$
$Q_{C_{10}}=Q_{C_{11}}$	$Q_{C_{11}}=Q$	$Q_{C_{10}}=Q_{C_{11}}$	$Q_{C_{11}}=Q$	$Q_{C_{10}}=Q_{C_{11}}$	$Q_{C_{11}}=Q$

**Table 2:** KVL to relate different $V_{C_{z}}$.

N=1		N=2		N→∞	
**Curr. Enters ** $N_{a}$	**Curr. Enters ** $N_{b}$	**Curr. Enters ** $N_{a}$	**Curr. Enters ** $N_{b}$	**Curr. Enters ** $N_{a}$	**Curr. Enters ** $N_{b}$
$V_{C_{1}}=V_{1o}$	$V_{C_{2}}=V_{1e}+V_{C_{1}}-\frac{Q_{C_{1}}}{C_{1}}$	$V_{C_{1}}=V_{1o}$	$V_{C_{2}}=V_{1e}+V_{C_{1}}-\frac{Q_{C_{1}}}{C_{1}}$	$V_{C_{1}}=V_{1o}$	$V_{C_{2}}=V_{1e}+V_{C_{1}}-\frac{Q_{C_{1}}}{C_{1}}$
$V_{C_{3}}=V_{C_{2}}-\frac{Q_{C_{2}}}{C_{2}}$	$V_{C_{4}}=V_{C_{3}}-\frac{Q_{C_{3}}}{C_{3}}$	$V_{C_{3}}=V_{p}+V_{C_{2}}-\frac{Q_{C_{2}}}{C_{2}}$	$V_{C_{4}}=V_{p}+V_{C_{3}}-\frac{Q_{C_{3}}}{C_{3}}$	$V_{C_{3}}=V_{p}+V_{C_{2}}-\frac{Q_{C_{2}}}{C_{2}}$	$V_{C_{4}}=V_{p}+V_{C_{3}}-\frac{Q_{C_{3}}}{C_{3}}$
$V_{C_{5}}=V_{C_{4}}-\frac{Q_{C_{4}}}{C_{4}}$	$V_{C_{6}}=V_{C_{5}}-\frac{Q_{C_{5}}}{C_{5}}$	$V_{C_{5}}=V_{p}+V_{C_{4}}-\frac{Q_{C_{4}}}{C_{4}}-V_{C_{3}}$	$V_{C_{6}}=V_{C_{5}}+V_{C_{4}}-\frac{Q_{C_{4}}}{C_{4}}$	$V_{C_{5}}=V_{p}+V_{C_{4}}-\frac{Q_{C_{4}}}{C_{4}}$	$V_{C_{6}}=V_{p}+V_{C_{5}}-\frac{Q_{C_{5}}}{C_{5}}$
$V_{C_{7}}=V_{C_{6}}-\frac{Q_{C_{6}}}{C_{6}}$	$V_{C_{8}}=V_{C_{7}}-\frac{Q_{C_{7}}}{C_{7}}$	$V_{C_{7}}=V_{C_{5}}+V_{C_{6}}-\frac{Q_{C_{6}}}{C_{6}}$	$V_{C_{8}}=V_{C_{5}}+V_{C_{7}}-\frac{Q_{C_{7}}}{C_{7}}$	$V_{C_{7}}=V_{p}+V_{C_{6}}-\frac{Q_{C_{6}}}{C_{6}}$	$V_{C_{8}}=V_{p}+V_{C_{7}}-\frac{Q_{C_{7}}}{C_{7}}$
$V_{C_{9}}=V_{C_{8}}-\frac{Q_{C_{8}}}{C_{8}}$	$V_{C_{10}}=V_{C_{9}}-\frac{Q_{C_{9}}}{C_{9}}$	$V_{C_{9}}=V_{C_{5}}+V_{C_{8}}-\frac{Q_{C_{8}}}{C_{8}}-V_{C_{7}}$	$V_{C_{10}}=V_{C_{9}}+V_{C_{8}}-\frac{Q_{C_{8}}}{C_{8}}$	$V_{C_{9}}=V_{p}+V_{C_{8}}-\frac{Q_{C_{8}}}{C_{8}}$	$V_{C_{10}}=V_{p}+V_{C_{9}}-\frac{Q_{C_{9}}}{C_{9}}$
$V_{C_{11}}=V_{C_{10}}-\frac{Q_{C_{10}}}{C_{10}}$		$V_{C_{11}}=V_{C_{9}}+V_{C_{10}}-\frac{Q_{C_{10}}}{C_{10}}$		$V_{C_{11}}=V_{p}+V_{C_{10}}-\frac{Q_{C_{10}}}{C_{10}}$	

**Table 3:** $V_{C_{o}}$ of 12P(N)OD-CVMR obtained through KVL.

*N*	$V_{C_{o}}$
1	$V_{1e}+V_{C_{1}}-\frac{Q_{C_{1}}}{C_{1}}+V_{C_{3}}-\frac{Q_{C_{3}}}{C_{3}}+V_{C_{5}}-\frac{Q_{C_{5}}}{C_{5}}+V_{C_{7}}-\frac{Q_{C_{7}}}{C_{7}}+V_{C_{9}}-\frac{Q_{C_{9}}}{C_{9}}+V_{C_{11}}-\frac{Q_{C_{11}}}{C_{11}}$
2	$V_{1e}+V_{C_{3}}-\frac{Q_{C_{3}}}{C_{3}}+V_{C_{7}}-\frac{Q_{C_{7}}}{C_{7}}+V_{C_{11}}-\frac{Q_{C_{11}}}{C_{11}}$
∞	$V_{1e}+V_{C_{11}}-\frac{Q_{C_{11}}}{C_{11}}$

**Table 4:** $q_{C_{z}}=\frac{Q_{C_{z}}}{Q}$, $V_{C_{z}}$, $V_{C_{z,M}}$, and $V_{C_{z,L}}$ of flying Caps. in 12P1OD-CVMR.

Cap.	12P1OD-CVMR			
$C_{z}$	$q_{C_{z}}$	$V_{C_{z}}$	$V_{C_{z,M}}$	$V_{C_{z,L}}$
$C_{1}$	6	$V_{1o}$	$V_{1o}-\frac{3Q}{C}$	$V_{1o}-\frac{6Q}{C}$
$C_{2}$	5	$V_{1o}+V_{1e}-\frac{6Q}{C}$	$V_{1o}+V_{1e}-\frac{17Q}{2C}$	$V_{1o}+V_{1e}-\frac{11Q}{C}$
$C_{3}$	5	$V_{1o}+V_{1e}-\frac{11Q}{C}$	$V_{1o}+V_{1e}-\frac{27Q}{2C}$	$V_{1o}+V_{1e}-\frac{16Q}{C}$
$C_{4}$	4	$V_{1o}+V_{1e}-\frac{16Q}{C}$	$V_{1o}+V_{1e}-\frac{18Q}{C}$	$V_{1o}+V_{1e}-\frac{20Q}{C}$
$C_{5}$	4	$V_{1o}+V_{1e}-\frac{20Q}{C}$	$V_{1o}+V_{1e}-\frac{22Q}{C}$	$V_{1o}+V_{1e}-\frac{24Q}{C}$
$C_{6}$	3	$V_{1o}+V_{1e}-\frac{24Q}{C}$	$V_{1o}+V_{1e}-\frac{51Q}{2C}$	$V_{1o}+V_{1e}-\frac{27Q}{C}$
$C_{7}$	3	$V_{1o}+V_{1e}-\frac{27Q}{C}$	$V_{1o}+V_{1e}-\frac{57Q}{2C}$	$V_{1o}+V_{1e}-\frac{30Q}{C}$
$C_{8}$	2	$V_{1o}+V_{1e}-\frac{30Q}{C}$	$V_{1o}+V_{1e}-\frac{31Q}{C}$	$V_{1o}+V_{1e}-\frac{32Q}{C}$
$C_{9}$	2	$V_{1o}+V_{1e}-\frac{32Q}{C}$	$V_{1o}+V_{1e}-\frac{33Q}{C}$	$V_{1o}+V_{1e}-\frac{34Q}{C}$
$C_{10}$	1	$V_{1o}+V_{1e}-\frac{34Q}{C}$	$V_{1o}+V_{1e}-\frac{69Q}{2C}$	$V_{1o}+V_{1e}-\frac{35Q}{C}$
$C_{11}$	1	$V_{1o}+V_{1e}-\frac{35Q}{C}$	$V_{1o}+V_{1e}-\frac{71Q}{2C}$	$V_{1o}+V_{1e}-\frac{36Q}{C}$
$C_{o}$	1	$6V_{1o}+6V_{1e}-\frac{146Q}{C}$	$6V_{1o}+6V_{1e}-\frac{293Q}{2C}$	$6V_{1o}+6V_{1e}-\frac{147Q}{C}$

**Table 5:** $q_{C_{z}}=\frac{Q_{C_{z}}}{Q}$, $V_{C_{z}}$, $V_{C_{z,M}}$, and $V_{C_{z,L}}$ of flying Caps. in 12P2OD-CVMR.

Cap.	12P2OD-CVMR			
$C_{z}$	$q_{C_{z}}$	$V_{C_{z}}$	$V_{C_{z,M}}$	$V_{C_{z,L}}$
$C_{1}$	1	$V_{1o}$	$V_{1o}-\frac{Q}{2C}$	$V_{1o}-\frac{Q}{C}$
$C_{2}$	1	$V_{1o}+V_{1e}-\frac{Q}{C}$	$V_{1o}+V_{1e}-\frac{3Q}{2C}$	$V_{1o}+V_{1e}-\frac{2Q}{C}$
$C_{3}$	5	$2V_{1o}+V_{1e}-\frac{2Q}{C}$	$2V_{1o}+V_{1e}-\frac{9Q}{2C}$	$2V_{1o}+V_{1e}-\frac{7Q}{C}$
$C_{4}$	4	$2V_{1o}+2V_{1e}-\frac{7Q}{C}$	$2V_{1o}+2V_{1e}-\frac{9Q}{C}$	$2V_{1o}+2V_{1e}-\frac{11Q}{C}$
$C_{5}$	1	$V_{1o}+V_{1e}-\frac{9Q}{C}$	$V_{1o}+V_{1e}-\frac{19Q}{2C}$	$V_{1o}+V_{1e}-\frac{10Q}{C}$
$C_{6}$	1	$V_{1o}+V_{1e}-\frac{10Q}{C}$	$V_{1o}+V_{1e}-\frac{21Q}{2C}$	$V_{1o}+V_{1e}-\frac{11Q}{C}$
$C_{7}$	3	$2V_{1o}+2V_{1e}-\frac{20Q}{C}$	$2V_{1o}+2V_{1e}-\frac{43Q}{2C}$	$2V_{1o}+2V_{1e}-\frac{23Q}{C}$
$C_{8}$	2	$2V_{1o}+2V_{1e}-\frac{23Q}{C}$	$2V_{1o}+2V_{1e}-\frac{24Q}{2C}$	$2V_{1o}+2V_{1e}-\frac{25Q}{C}$
$C_{9}$	1	$V_{1o}+V_{1e}-\frac{14Q}{C}$	$V_{1o}+V_{1e}-\frac{29Q}{2C}$	$V_{1o}+V_{1e}-\frac{15Q}{C}$
$C_{10}$	1	$V_{1o}+V_{1e}-\frac{15Q}{C}$	$V_{1o}+V_{1e}-\frac{31Q}{2C}$	$V_{1o}+V_{1e}-\frac{16Q}{C}$
$C_{11}$	1	$2V_{1o}+2V_{1e}-\frac{30Q}{C}$	$2V_{1o}+2V_{1e}-\frac{61Q}{2C}$	$2V_{1o}+2V_{1e}-\frac{31Q}{C}$
$C_{o}$	1	$6V_{1o}+6V_{1e}-\frac{61Q}{C}$	$6V_{1o}+6V_{1e}-\frac{123Q}{2C}$	$6V_{1o}+6V_{1e}-\frac{62Q}{C}$

**Table 6:** $q_{C_{z}}=\frac{Q_{C_{z}}}{Q}$, $V_{C_{z}}$, $V_{C_{z,M}}$, and $V_{C_{z,L}}$ of flying Caps. in 12PDOD-CVMR.

Cap.	12PDOD-CVMR			
$C_{z}$	$q_{C_{z}}$	$V_{C_{z}}$	$V_{C_{z,M}}$	$V_{C_{z,L}}$
$C_{1}$	1	$V_{1o}$	$V_{1o}-\frac{Q}{2C}$	$V_{1o}-\frac{Q}{C}$
$C_{2}$	1	$V_{1o}+V_{1e}-\frac{Q}{C}$	$V_{1o}+V_{1e}-\frac{3Q}{2C}$	$V_{1o}+V_{1e}-\frac{2Q}{C}$
$C_{3}$	1	$2V_{1o}+V_{1e}-\frac{2Q}{C}$	$2V_{1o}+V_{1e}-\frac{5Q}{2C}$	$2V_{1o}+V_{1e}-\frac{3Q}{C}$
$C_{4}$	1	$2V_{1o}+2V_{1e}-\frac{3Q}{C}$	$2V_{1o}+2V_{1e}-\frac{7Q}{2C}$	$2V_{1o}+2V_{1e}-\frac{4Q}{C}$
$C_{5}$	1	$3V_{1o}+2V_{1e}-\frac{4Q}{C}$	$3V_{1o}+2V_{1e}-\frac{9Q}{2C}$	$3V_{1o}+2V_{1e}-\frac{5Q}{C}$
$C_{6}$	1	$3V_{1o}+3V_{1e}-\frac{5Q}{C}$	$3V_{1o}+3V_{1e}-\frac{11Q}{2C}$	$3V_{1o}+3V_{1e}-\frac{6Q}{C}$
$C_{7}$	1	$4V_{1o}+3V_{1e}-\frac{6Q}{C}$	$4V_{1o}+3V_{1e}-\frac{13Q}{2C}$	$4V_{1o}+3V_{1e}-\frac{7Q}{C}$
$C_{8}$	1	$4V_{1o}+4V_{1e}-\frac{7Q}{C}$	$4V_{1o}+4V_{1e}-\frac{15Q}{2C}$	$4V_{1o}+4V_{1e}-\frac{8Q}{C}$
$C_{9}$	1	$5V_{1o}+4V_{1e}-\frac{8Q}{C}$	$5V_{1o}+4V_{1e}-\frac{17Q}{2C}$	$5V_{1o}+4V_{1e}-\frac{9Q}{C}$
$C_{10}$	1	$5V_{1o}+5V_{1e}-\frac{9Q}{C}$	$5V_{1o}+5V_{1e}-\frac{19Q}{2C}$	$5V_{1o}+5V_{1e}-\frac{10Q}{C}$
$C_{11}$	1	$6V_{1o}+5V_{1e}-\frac{10Q}{C}$	$6V_{1o}+5V_{1e}-\frac{21Q}{2C}$	$6V_{1o}+5V_{1e}-\frac{11Q}{C}$
$C_{o}$	1	$6V_{1o}+6V_{1e}-\frac{11Q}{C}$	$6V_{1o}+6V_{1e}-\frac{23Q}{2C}$	$6V_{1o}+6V_{1e}-\frac{12Q}{C}$

**Table 7:** Effective capacitance seen at the input node of 12P(N)OD-CVMR.

N=1	Figure 2b	$C_{a}$	$\left(C_{11}+C_{10}\right)\left|\right|\left(C_{9}+C_{8}\right)\left|\right|\left(C_{7}+C_{6}\right)\left|\right|\left(C_{5}+C_{4}\right)\left|\right|\left(C_{3}+C_{2}\right)\left|\right|C_{1}$	$\frac{2C}{7}$
	Figure 2c	$C_{b}$	$\left\{\left(C_{o}\left|\right|C_{11}\right)\left|\right|\left(C_{10}+C_{9}\right)\left|\right|\left(C_{8}+C_{7}\right)\left|\right|\left(C_{6}+C_{5}\right)\left|\right|\left(C_{4}+C_{3}\right)+C_{2}\right\}\left|\right|C_{1}$	$\frac{5C}{9}$
N=2	Figure 3b	$C_{a}$	$\left[\left\{\left(\left(\left(\left(\left(\left(C_{11}\left|\right|C_{10}\right)+C_{9}\right)\left|\right|C_{8}\right)+C_{6}\right)\left|\right|C_{7}\right)+C_{5})||C_{4}\right)+C_{2}\right\}\left|\right|C_{3}\right]+C_{1}$	$\frac{144C}{89}$
	Figure 3c	$C_{b}$	$\left[\left\{C_{o}\left|\right|C_{11}\left|\right|\left(C_{8}+C_{7}\right)+C_{4}\right\}\left|\right|C_{3}\right]+\left(C_{2}\left|\right|C_{1}\right)$	$\frac{13C}{12}$
N→∞	Figure 4b	$C_{a}$	$\left(C_{11}\left|\right|C_{10}\right)+\left(C_{9}\left|\right|C_{8}\right)+\left(C_{7}\left|\right|C_{6}\right)+\left(C_{5}\left|\right|C_{4}\right)+\left(C_{3}+C_{2}\right)+C_{1}$	$\frac{7C}{2}$
	Figure 4c	$C_{b}$	$\left(C_{o}\left|\right|C_{11}\right)+\left(C_{10}\left|\right|C_{9}\right)+\left(C_{8}\left|\right|C_{7}\right)+\left(C_{6}\left|\right|C_{5}\right)+\left(C_{4}\left|\right|C_{3}\right)+\left(C_{2}\left|\right|C_{1}\right)$	3C

**Table 8:** Approximate theoretical analysis of the UHGH converters.

Conv.	$G=\frac{V_{o}}{V_{\mathrm{bat}}}$	Avg. Curr. Ind.	Avg. Volt. Cap.	SW. Volt.	HVW Volt.
Family1					
Figure 5a	$\frac{W_{1}T}{2(1-D)}$	$I_{L}=I_{o}G$	NA	$V_{S_{l1}}=V_{S_{l2}}=V_{S_{l3}}=V_{S_{l4}}=\frac{V_{bat}}{2-2D}$	$V_{1o}=V_{1e}=\frac{W_{1}}{2(1-D)}$
Figure 5b	$\frac{W_{1}T}{2(1-D)}$	$I_{L}=I_{o}G$	NA	$V_{S_{l1}}=V_{S_{l2}}=V_{S_{l3}}=V_{S_{l4}}=\frac{V_{bat}}{2-2D}$	$V_{1o}=V_{1e}=\frac{W_{1}}{2(1-D)}$
Family2					
Figure 6a	$\frac{W_{1}TD}{\left(1-D\right)}$	$I_{ma}=I_{mb}=\frac{I_{o}G}{2D}$	$V_{C_{al}}=V_{C_{bl}}=\frac{DV_{bat}}{1-D}$	$V_{D_{a1}}=V_{S_{a2}}=V_{D_{b1}}=V_{S_{b2}}=\frac{V_{bat}}{1-D}$ $V_{D_{a3}}=V_{D_{b3}}=W_{1}V_{bat}$ $V_{S_{a5}}=V_{S_{b5}}=\frac{W_{1}DV_{bat}}{1-D}$	$V_{1o}=V_{1e}=\frac{W_{1}D}{\left(1-D\right)}$
Figure 6b	$\frac{(1+W_{1})TD}{\left(1-D\right)}$	$I_{La}=I_{Lb}=\frac{I_{o}G}{2}$ $I_{ma}=I_{mb}=\frac{I_{o}GD}{2-2D}$	$V_{C_{al}}=V_{C_{bl}}=\frac{DV_{bat}}{1-D}$	$V_{D_{a1}}=V_{S_{a2}}=V_{D_{b1}}=V_{S_{b2}}=\frac{V_{bat}}{1-D}$ $V_{D_{a3}}=V_{D_{b3}}=\left(W_{1}-\frac{D}{1-D}\right)V_{bat}$ $V_{S_{a5}}=V_{S_{b5}}=\frac{\left(1+W_{1}D\right)V_{bat}}{1-D}$	$V_{1o}=V_{1e}=\frac{(W_{1}+1)D}{\left(1-D\right)}$
Family3					
Figure 7a	$\frac{\left(1+W_{1}T\right)}{\left(1-D\right)}$	$I_{ma}=I_{mb}=\frac{I_{o}G}{D}$	$V_{C_{l}}=\frac{V_{bat}}{1-D}$	$V_{D_{a1}}=V_{S_{a2}}=V_{D_{b1}}=V_{S_{b2}}=\frac{V_{bat}}{1-D}$	$V_{1o}=V_{1e}=\frac{W_{1}}{\left(1-D\right)}$
Figure 7b	$\frac{\left(1+W_{1}T\right)}{\left(1-D\right)}$	$I_{La}=I_{Lb}=\frac{I_{o}G}{2D}$	$V_{C_{l}}=\frac{V_{bat}}{1-D}$	$V_{D_{a1}}=V_{S_{a2}}=V_{D_{b1}}=V_{S_{b2}}=\frac{V_{bat}}{1-D}$	$V_{1o}=V_{1e}=\frac{W_{1}}{\left(1-D\right)}$
Family4					
Figure 8a	$\frac{\left(1+0.5*W_{1}T\right)}{\left(1-D\right)}$	$I_{m}=I_{o}G$	$V_{C_{l}}=\frac{V_{bat}}{1-D}$	$V_{D_{a1}}=V_{S_{a2}}=V_{D_{b1}}=V_{S_{b2}}=\frac{V_{bat}}{1-D}$	$V_{1o}=V_{1e}=\frac{W_{1}}{2(1-D)}$
Figure 8b	$\frac{\left(1+0.5*W_{1}T\right)}{\left(1-D\right)}$	$I_{L}=I_{o}G$ $I_{m}=0$	$V_{C_{l1}}=V_{bat}$ $V_{C_{l2}}=\frac{V_{bat}}{1-D}$	$V_{D_{a1}}=V_{S_{a2}}=V_{D_{b1}}=V_{S_{b2}}=\frac{V_{bat}}{1-D}$	$V_{1o}=V_{1e}=\frac{W_{1}}{2(1-D)}$

**Table 9:** Simulation Results of the HGSME of the UHGH converters.

Conv.	Ind. (uH)	Avg. Ind. Curr. (A)	Cap. (uF)	Avg. Cap. Volt. (V)
Family1				
Figure 5a	L=7.5	$I_{L}=5.4$	NA	NA
Figure 5b	L=7.5	$I_{L}=5.4$	NA	NA
Family2				
Figure 6a	$L_{ma}=L_{mb}=25$	$I_{ma}=I_{mb}=3.5$	$C_{al}=C_{bl}=22$	$V_{C_{al}}=V_{C_{bl}}=11$
Figure 6b	$L_{a}=L_{b}=50$ $L_{ma}=L_{mb}=50$	$I_{L_{a}}=I_{L_{b}}=2.8$ $I_{ma}=I_{mb}=1$	$C_{a2}=C_{b2}=22$ $C_{a1}=C_{b1}=220$	$V_{C_{a1}}=V_{C_{b1}}=3.7$ $V_{C_{a2}}=V_{C_{b2}}=26$
Family3				
Figure 7a	$L_{ma}=L_{mb}=50$	$I_{ma}=I_{mb}=2.5$	$C_{z}=0.002$	$V_{C_{l}}=160$
Figure 7b	$L_{a}=L_{b}=25$	$I_{L_{a}}=I_{L_{b}}=2.75$	$C_{z}=0.2$	$V_{C_{l}}=15$
Family4				
Figure 8a	$L_{m}=50$	$I_{m}=5.5$	$C_{z}=0.022$	$V_{C_{l}}=93$
Figure 8b	$L=L_{m}=50$	$I_{L}=5.5$; $I_{m}=0$	$C_{li}=C_{l2}=0.22$	$V_{C_{1}}=3.7$; $V_{C_{2}}=11$

**Table 10:** Composition of the circuit.

Part Name	Part Number	Rating	PPU ($)	WPU (Oz)
Micro-controller	TMS320f28379D		14.56	0.09
Half-bridge driver	UCC27201DR	120 V, 3 A	0.756	0.00256
Low-side driver	IX4427NTR	34 V, 1.5 A	0.35	0.019
Decoup. Cap.	C0805C105K3RAC7210	25 V, 1 uF	0.22	0.0002
Boot-strap capacitor	C0805C104K3RACTU	25 V, 0.1 uF	0.037	0.0002
$C_{al},C_{bl}$	C2012X5R1V226M125AC	35 V, 22 uF	0.285	0.0002
$S_{a1},S_{a2},S_{b1},S_{b2}$	SIRA20DP-T1-RE3	25 V, 63 A	0.56	0.018
$W_{a},W_{b}$	XF0757-EF25R-A	1:100	1.85	1.8
$D_{a3},D_{b3}$	GAP3SLT33-214	3.3 kV, 0.3 A	7.3	0.0035
$S_{a5},S_{b5}$	IXTY02N120P	1.2 kV, 0.2 A	0.66	0.08
$D_{1},D_{2},\ldots ,D_{12}$	GP02-40-E3/73	5 kV, 0.25 A	0.14	0.012
$C_{o}$	HVCC153Y6P202MEAX	2 nF, 15 kV	2.17	0.237
$C_{1},C_{2}$	2225WC223KAT1A	22 nF, 2.5 kV	1.66	0.02481
$C_{3},C_{4}$	HV2225Y332KXmathV	3.3 nF, 5 kV	1.14	0.044446
$C_{5},C_{6},\ldots ,C_{11}$	HVCC153Y6P202MEAX	2 nF, 15 kV	2.17	1.659

PPU: Price Per Unit; WPU: Weight Per Unit.

**Table 11:** Comparison with [33].

Conv. Capabilities	[33]	This
Max. Op. Volt. (kV)	5	7
Max. Op. Curr. (mA)	0.15	5
Max. Op. Pow. (W)	0.75	25
Pow. Density (W/in^3^)	0.06	0.4
Pow. Density (W/g)	0.0125	0.15
Cost (USD)	390	70
Pow. Density (W/$)	0.002	0.36

For both assemblies, the volume of the entire packaging is considered, and DSP cost is excluded.

**Table 12:** Qualitative comparison of the UHGH converters.

Figure	S	D	W	CW	TR	C	CS	H	I	CI
Family1										
Figure 5a	2	12	4	1×3	110	12	2	0	Y	Y
Figure 5b	4	12	2	1×2	110	12	2	2	Y	Y
Family2										
Figure 6a	4	16	4	2×2	75	14	2	0	Y	N
Figure 6b	4	16	6	2×2	72	16	2	0	N	Y
Family3										
Figure 7a	2	14	4	2×2	55	14	2	0	N	N
Figure 7b	2	14	4	1×2	55	13	2	0	N	Y
Family4										
Figure 8a	1	13	2	1×2	110	13	1	0	N	N
Figure 8b	2	12	3	1×2	111	14	1	1	N	Y

S—No. of switches; D—No. of diodes; W—No. of Windings; CW—Number of coupled windings; TR—Turn ratio of coupled inductor or transformer; C—No. of capacitors; CS—No. of control signals; H—No. of high side driver; I—Presence of isolation (Yes or No); CI—Whether the input current is continuous or not.

**Table 13:** Comparison of output voltages of different HGSMEs and $v_{C_{z}}$s of different CVMRs of the UHGH converters.

Conv.	*N*	$V_{1o}$	$V_{1e}$	$V_{C_{1}}$	$V_{C_{2}}$	$V_{C_{3}}$	$V_{C_{4}}$	$V_{C_{5}}$	$V_{C_{6}}$	$V_{C_{7}}$	$V_{C_{8}}$	$V_{C_{9}}$	$V_{C_{10}}$	$V_{C_{11}}$	$V_{C_{o}}$
Families 1 (Figure 5), 2 (Figure 6) and 3 (Figure 7)	∞	0.8	0.8	0.8	1.6	2.4	3.2	4	4.8	5.6	6.4	7.2	8	8.8	9.6
	2	0.9	0.9	0.9	1.8	2.5	3.5	1.5	1.5	3	3	1.4	1.4	2.7	9.3
	1	0.9	0.9	0.9	1.7	1.6	1.6	1.6	1.6	1.6	1.6	1.6	1.6	1.6	9.6
Family 4 (Figure 8)	∞	0.4	1.4	0.4	1.6	2	3.2	3.6	4.8	5.2	6.4	6.8	8	8.4	9.6
	2	0.5	1.3	0.5	1.8	2.4	3.4	1.6	1.6	3.1	3	1.4	1.4	2.8	9.5
	1	0.4	1.2	0.4	1.6	1.6	1.6	1.6	1.6	1.6	1.6	1.6	1.5	1.5	9.6

## Data Availability

Not applicable.

## Abbreviations

LV	Low Voltage
HV	High Voltage
HVW	High Voltage Winding
LVW	Low Voltage Winding
HGSME	High-Gain Switched Magnetic Element
UHGH	Ultra-High Gain Hybrid
HASEL	Hydraulically Amplified Self-healing Electrostatic
